# Associations between habitual diet, metabolic disease, and the gut microbiota using latent Dirichlet allocation

**DOI:** 10.1186/s40168-020-00969-9

**Published:** 2021-03-16

**Authors:** Taylor A. Breuninger, Nina Wawro, Jakob Breuninger, Sandra Reitmeier, Thomas Clavel, Julia Six-Merker, Giulia Pestoni, Sabine Rohrmann, Wolfgang Rathmann, Annette Peters, Harald Grallert, Christa Meisinger, Dirk Haller, Jakob Linseisen

**Affiliations:** 1 Independent Research Unit Clinical Epidemiology, Helmholtz Zentrum München, German Research Center for Environmental Health (GmbH), Ingolstädter Landstr. 1, 85764 Neuherberg, Germany; 2 Ludwig-Maximilians-Universität München, UNIKA-T Augsburg, Neusässer Str. 47, 86156 Augsburg, Germany; 3 Delicious Data GmbH, Lichtenbergstr. 8, 85748 Garching, Germany; 4 Technische Universität München, Gregor-Mendel-Str. 2, 85354 Freising, Germany; 5 ZIEL - Institute for Food & Health, Technische Universität München, Weihenstephaner Berg 3, 85354 Freising, Germany; 6 Functional Microbiome Research Group, Institute of Medical Microbiology, RWTH University Hospital, Pauwelsstrasse 30, 52074 Aachen, Germany; 7 Institute of Epidemiology, Helmholtz Zentrum München, German Research Center for Environmental Health (GmbH), Ingolstädter Landstr. 1, 85764 Neuherberg, Germany; 8 Division of Chronic Disease Epidemiology, Epidemiology, Biostatistics and Prevention Institute, University of Zurich, Hirschengraben 84, CH-8001 Zurich, Switzerland; 9 Institute for Biometrics and Epidemiology, Deutsches Diabetes-Zentrum (DDZ), Auf’m Hennekamp 65, 40225 Düsseldorf, Germany

**Keywords:** *enable*-Cluster, 16S rRNA gene sequencing, Nutrition, Dietary intake, Diabetes, Serum lipids, Obesity, Hypertension

## Abstract

**Background:**

The gut microbiome impacts human health through various mechanisms and is involved in the development of a range of non-communicable diseases. Diet is a well-known factor influencing microbe-host interaction in health and disease. However, very few findings are based on large-scale analysis using population-based studies. Our aim was to investigate the cross-sectional relationship between habitual dietary intake and gut microbiota structure in the Cooperative Health Research in the Region of Augsburg (KORA) FF4 study.

**Results:**

Fecal microbiota was analyzed using 16S rRNA gene amplicon sequencing. Latent Dirichlet allocation (LDA) was applied to samples from 1992 participants to identify 20 microbial subgroups within the study population. Each participant’s gut microbiota was subsequently described by a unique composition of these 20 subgroups. Associations between habitual dietary intake, assessed via repeated 24-h food lists and a Food Frequency Questionnaire, and the 20 subgroups, as well as between prevalence of metabolic diseases/risk factors and the subgroups, were assessed with multivariate-adjusted Dirichlet regression models. After adjustment for multiple testing, eight of 20 microbial subgroups were significantly associated with habitual diet, while nine of 20 microbial subgroups were associated with the prevalence of one or more metabolic diseases/risk factors. Subgroups 5 (*Faecalibacterium*, *Lachnospiracea incertae sedis*, *Gemmiger*, *Roseburia*) and 14 (*Coprococcus*, *Bacteroides*, *Faecalibacterium*, *Ruminococcus*) were particularly strongly associated with diet. For example, participants with a high probability for subgroup 5 were characterized by a higher Alternate Healthy Eating Index and Mediterranean Diet Score and a higher intake of food items such as fruits, vegetables, legumes, and whole grains, while participants with prevalent type 2 diabetes mellitus were characterized by a lower probability for subgroup 5.

**Conclusions:**

The associations between habitual diet, metabolic diseases, and microbial subgroups identified in this analysis not only expand upon current knowledge of diet-microbiota-disease relationships, but also indicate the possibility of certain microbial groups to be modulated by dietary intervention, with the potential of impacting human health. Additionally, LDA appears to be a powerful tool for interpreting latent structures of the human gut microbiota. However, the subgroups and associations observed in this analysis need to be replicated in further studies.

**Video abstract**

**Supplementary Information:**

The online version contains supplementary material available at 10.1186/s40168-020-00969-9.

## Background

The last two decades of research have extensively studied the role of the human gut microbiome in host health [1]. The gut microbiome can be considered as a metabolically active organ that produces a multitude of metabolites that either positively or negatively affect human physiology [2]. Additionally, the composition and diversity of the gut microbiota have been associated with a wide range of diseases, ranging from gastrointestinal disorders such as *Clostridium difficile* infection to conditions such as type 2 diabetes mellitus (T2DM), cardiovascular disease, depression, and rheumatoid arthritis [3–7].

In addition to the many ways the gut microbiome influences its host, it has become clear that a variety of different factors influence the microbiome itself, including genetics, geographic environment, medication (especially antibiotics), nutrition, age, lifestyle, and mode of delivery at birth [8–13]. The number of modifiable factors associated with the gut microbiome, combined with its association with many disease states, presents the tempting possibility of influencing the development or progression of disease by modifying the gut microbiome. Yet this is complicated by wide interindividual variability [14, 15]. Furthermore, compositional changes do not necessarily translate into functional alterations, as different bacteria may perform similar functions [14]. As a result, it has so far proven difficult to characterize exactly what “healthy” microbiomes are [15].

Nutrition in particular is accepted as a major modifiable factor of the gut microbiome, yet it has also been independently associated with many of the same diseases that the gut microbiome is proposed to modulate [16–20]. In turn, the microbiota can also influence the absorption and metabolism of nutrients and other food components [15, 21]. This makes the task of unraveling the true nature of these associations challenging. While intense interest has produced a wealth of information on modifying the microbiome through nutrition, much of this still needs to be confirmed in large-scale, population-based cohorts.

We previously characterized the compositional and functional profiles of the prospective cohort Cooperative Health Research in the Region of Augsburg (KORA) and identified bacterial signatures of the development of T2DM [22]. Our aim in the present analysis was to evaluate the cross-sectional relationship between gut microbiota structure and habitual dietary intake in this large, population-based cohort. To this end, we applied an unsupervised machine learning method to identify latent structures (microbial subgroups) within the data and associations between habitual diet and/or metabolic diseases and these subgroups. In two subanalyses, we used the bacterial risk signatures of T2DM we previously established in this population and enterotype-like clusters to demonstrate the ability of LDA to identify latent features of the microbiota that would inherently be missed by other methods.

## Methods

This analysis is reported according to the “Strengthening the Reporting of Observational Studies in Epidemiology - Nutritional Epidemiology (STROBE-nut)” recommendations [23].

### Study population

The data used in this analysis originated from the KORA FF4 study (2013/2014), which is the second in a series of follow-up surveys of the original KORA S4 study, conducted from 1999 to 2001. KORA S4 is a population-based study that included 4261 participants recruited from the southern German city of Augsburg and its two surrounding counties. The FF4 survey included 2279 individuals from the original S4 study, ranging from 38 to 88 years old. Details concerning the design of the KORA studies have been published previously [24].

### Collection and processing of biosamples

Participants in KORA FF4 collected a single stool sample according to paper-based instructions at home. They were also given instructions over the phone and were mailed a sterile stool collection kit. One spoonful taken from two different areas of the stool specimen was to be deposited into a tube containing 5 ml of DNA stabilizer (Stratec DNA Stool Stabilizer, No. 1038111100). The stool sample was to be collected on the morning of the study center visit and kept in the refrigerator until the appointment. If this was not possible, the stool sample could be mailed in afterward. Additionally, a short questionnaire was to be filled out regarding details of the sample collection (consistency, problems, time, storage conditions, etc.). After transport to the study center, samples were frozen at − 80 °C until further processing. Any participants who took antibiotics in the previous 2 months were excluded. Further details of the collection procedure and handling until analysis were described previously [25]. Samples were submitted from 2076 participants.

### 16S rRNA gene amplicon sequencing

Preparation and analysis of the gut microbiota samples were done as described in Reitmeier et al. [22]. Briefly, the metagenomic DNA was isolated from 600 μl of stool in DNA stabilizer solution using a modified version of the protocol by Godon et al. [26]. A FastPrep-24 instrument fitted with a cooling adapter and 0.1-mm glass beads was used to lyse microbial cells. NucleoSpin gDNA columns (Machery-Nagel, No. 740230.250) were used to purify the DNA. A robotic liquid handler was used for all pipetting steps after DNA extraction until sequencing to maximize reproducibility.

Polymerase chain reaction (PCR) runs were conducted in duplicates. The extracted DNA was diluted in PCR-grade water and 24 ng was used as a template for amplification (25 cycles) using a two-step process to minimize bias [27]. The V3/V4 regions of 16S rRNA genes were amplified using 341F-ovh and 785r-ovh primers [28]. During cleaning, PCR products were pooled using magnetic beads (Beckman Coulter). Fluorimetry was used to determine PCR fragment concentration, which was adjusted to 2 nM. An Illumina HiSeq was used to sequence multiplexed samples in paired-end mode (2 × 250 bp) using the Rapid v2 chemistry. Samples with a read count < 4700 (high-quality, chimera-checked) were re-sequenced on an Illumina MiSeq using v3. To identify potential inconsistencies between the platforms, identical samples were sequenced on both the HiSeq and MiSeq and were compared. No differences could be identified between the taxonomic compositions of the runs. Two negative controls (a PCR control without template DNA and a DNA extraction control containing 600 μl stool stabilizer but no sample) and one positive control (mock community; ZymoBIOMICS, No. D6300) were included in every batch of 45 samples (processed on a single 96-well plate) to control for artifacts.

### Analysis of amplicon sequences

The 16S rRNA amplicon reads were preprocessed using the UPARSE-based IMNGS platform [29, 30]. Chimeras were removed using UCHIME [31]. Five nucleotides on both the 5′ and 3′end were trimmed for each of the R1 and R2 reads, respectively. The quality trim score was 5 and expected number of errors across assembled reads was 1. Sequences were clustered into operational taxonomic units (OTUs) at 97% sequence identity using UPARSE v8.1.1861_i86 [30]. OTUs occurring at a relative abundance < 0.25% across all samples were removed to prevent the analysis of spurious OTUs [32]. Taxonomies were assigned with adequate confidence (> 80%) to a maximum of genus level using the RDP classifier version 2.11 and confirmed using the SILVA database version 132 [33]. The OTU table containing 2091 OTUs was then normalized by total count per column to account for differences in library size. Taxonomic classification at the species levels for relevant OTUs was assigned using EzBioCloud (version 20200513) where possible [34].

### Assessment of dietary intake

Habitual dietary intake was assessed in KORA FF4 participants using a two-step method combining information from up to three repeated 24-h food lists (24HFL) and one Food Frequency Questionnaire (FFQ) [35, 36]. The calculation of dietary intake in KORA FF4 is based on the estimation of consumption probability and consumption amount. Details have been published previously, but briefly, consumption probability is determined for each food item for each individual based on the 24HFLs and FFQ, while usual portion size for each item is estimated based on data from the Bavarian Food Consumption Survey II (BVS II) [37]. Consumption probability multiplied by consumption amount then results in the usual intake of each food item on any given day. Food items were then categorized into 16 food groups and 21 subgroups based on the European Prospective Investigation into Cancer and Nutrition (EPIC)-Soft classification scheme [38]. In addition to the standard food groups and subgroups that are specified by the EPIC-Soft criteria, the variable “whole grains” was created from the food items “whole grain bread,” “whole grain toast,” and “muesli.” The variable “refined grains” was constructed by subtracting intake of whole grains from the food group “grains and grain products.” For the purposes of this analysis, the food group “dairy” excludes cheese and yogurt, as they were investigated individually. Habitual nutrient intake was calculated based on usual food intake using the National Nutrient Database (Bundeslebensmittelschlüssel; BLS 3.02). Information about dietary supplement use was collected with the 24HFL, but was not included in the calculation of habitual dietary intake. Habitual dietary intake data was available for 1602 participants; however, both microbiota and diet information were available for only 1442 participants. Therefore, all nutrition-related analyses were limited to this sample size. The Alternate Healthy Eating Index 2010 (AHEI, modified to exclude trans fats) and Mediterranean Diet Score 2003 (MDS) were calculated for this subsample of 1442 individuals as performed by Wawro et al. [39–41].

### Assessment of diseases/risk factors and covariates

On the day of the study center visit, a face-to-face interview was conducted, which gathered information on age (years), sex (male, female), education level (< 13 and ≥ 13 years, including vocational training), leisure time physical activity (> 1 h/week in summer and winter), smoking habits (current, ex-, never), medical diagnoses, and medication use, among other variables. Trained examiners took anthropometric measurements in standardized fashion. Blood pressure was measured and a fasting blood sample was drawn for the assessment of serum LDL-c, HDL-c, total cholesterol, and triglycerides. A self-reported diagnosis of diabetes or the use of antidiabetic medication was confirmed with the participant’s treating physician. An oral glucose tolerance test was carried out in all participants who did not have an existing diagnosis of diabetes. Participants were categorized based on their glucose tolerance status according to the 2003 American Diabetes Association diagnostic criteria as either (1) normal, (2) prediabetes, (3) undiagnosed diabetes mellitus, (4) known T2DM, or (5) other/unknown [42]. Participants’ hypertension status was classified according to existing diagnosis, if any, their blood pressure reading (according to the 1999 International Society of Hypertension-World Health Organization diagnostic criteria (≥ 140/90 mmHg) [43]), and use of antihypertensive medication into five groups: (1) normal (participant is normotensive); (2) known hypertension, controlled (participant is aware of hypertension, takes antihypertensive medication, and blood pressure is < 140/90 mmHg); (3) known hypertension, uncontrolled (participant is aware of hypertension and takes medication, but blood pressure is ≥ 140/90 mmHg); (4) known hypertension, not treated (participant is aware of hypertension, does not take medication, and blood pressure is ≥ 140/90 mmHg); and (5) undetected hypertension (participant is unaware of hypertension, does not take medication, and blood pressure is ≥ 140/90 mmHg). A detailed description of the assessment of the variables used in this analysis has already been presented in previous papers [25, 44].

### Statistical analysis

#### Latent Dirichlet allocation

For the purposes of our analysis, it was necessary to perform either clustering or dimensionality reduction in order to reduce the number of OTUs from 2091 to a more practical number. The concept of “enterotypes,” typically a three-cluster solution, is probably the most commonly employed clustering strategy in regard to microbiome data [45]. However, the concept of only two to three clusters that can adequately describe any gut microbiota sample is very limited in its application and ability to describe interindividual differences, especially when it comes to microbiota structure in relation to health and disease. Therefore, we elected to implement latent Dirichlet allocation (LDA), a Bayesian probabilistic generative model proposed by Blei et al. in 2003, which is used to uncover latent structures present in unlabeled data [46]. This popular unsupervised machine learning method has been implemented most commonly in the field of natural language processing, where it can identify latent topics (e.g., “sports,” “politics,” “science”) present in a collection of documents. However, LDA has also been applied to a variety of biological data types, including population genetics data, protein sequence data, magnetic resonance imaging data, and microbiome data, where it can learn latent microbial subgroups [47–53].

Assuming there is a total number of *O* observations and *K* subgroups, the generative process modelled by LDA (described in relation to our analysis) assumes that the gut microbiota structure of each observation can be represented by a multinomial distribution, parametrized by ***θ***_***i***_, over latent subgroups, where ***θ***_***i***_ is drawn from a latent Dirichlet distribution parametrized by ***α***:

$$ {\boldsymbol{\theta}}_{\boldsymbol{i}}\sim \mathrm{Dir}\left(\boldsymbol{a}\right),\mathrm{where}\ i\in \left\{1,\dots, O\right\}. $$

In turn, each subgroup is characterized by a multinomial distribution, parametrized by ***ϕ***_***k***_, over the OTUs, where ***ϕ***_***k***_ is drawn from another latent Dirichlet distribution parametrized by ***β***:

$$ {\boldsymbol{\phi}}_{\boldsymbol{k}}\sim \mathrm{Dir}\left(\boldsymbol{\beta} \right),\mathrm{where}\ k\in \left\{1,\dots, K\right\} $$

Given a total number of observations *O* and a total number of reads *N*_*i*_ for an observation *i*, the generation of all reads, where each read *q*_*i,j*_ with the position, *i* and *j*, where *i* ∈ {1, …, *O*} and *j* ∈ {1, …, *N*_*i*_}, is modelled consecutively by sampling the subgroup *k*_*i,j*_ *~* Multinomial(***θ***_***i***_) and a corresponding read *q*_*i,j*_ ~ Multinomial(***ϕ***_***ki,j***_). In both cases, the Multinomial distributions refer to Multinomial distributions with one trial.

Given a fitted model, the variables ***θ***_***i***_ and ***ϕ***_***k***_ are of particular interest. For each observation, ***θ***_***i***_ is a vector of probabilities over all subgroups, the sum of which is 1. A high probability of a subgroup means that it contributes to a large part of the microbiota structure of that observation. Likewise, for each subgroup, ***ϕ***_***k***_ is a vector of probabilities over all OTUs, the sum of which is 1. In this case, a high probability of an OTU means that OTU contributes to a large part of that microbial subgroup.

When compared to traditional clustering or classification methods, ***θ***_***i***_ can also be seen as fractional membership, meaning each observation’s microbiota structure can be described by a unique composition of several different microbial subgroups. For example, one observation may have a 45% probability for one subgroup, a 20% probability for a second subgroup, and a 5% probability for 7 more subgroups; another may have a 10% probability for 10 different subgroups. Likewise, each subgroup has a different probability of containing each of the 1713 OTUs. While fractional membership can also be achieved by fuzzy clustering methods, LDA differs from fuzzy clustering as well in that it learns patterns of co-occurrences of OTUs (***ϕ***_***k***_) rather than clustering observations based on distance measures. This means that each subgroup represents a group of microbes that tend to appear together, due to similar environmental requirements, functions, or because they are modulated by a shared external factor.

#### Calculation of microbial subgroups

Microbiota data were available for 2033 participants. All participants who reported taking systemic antibiotics in the previous 2 months were excluded (*n* = 41), leaving 1992 participants available for analysis.

After quality controls and reads processing, 2091 OTUs were kept for analysis. Before performing LDA, the OTU table was filtered so that only OTUs occurring at a relative abundance > 0.1% and 1% prevalence were included in order to reduce sparsity in the data set, resulting in remaining 1713 OTUs. A relatively low cutoff was chosen due to the nature of the method. LDA is designed to process data sets with many words (OTUs). Additionally, because part of our goal was to identify associations between the gut microbiota and diseases, removing OTUs present only across e.g. less than 10% of samples could potentially result in the loss of OTUs present only in a certain disease state.

The LDA model was fitted using Gibbs’ sampling with the R package *MetaTopics* version 1.0 [54]*.* As this package requires the input matrix to contain count data in the form of integers, and our OTU table consists of normalized counts that add up to 1 for each participant, we multiplied the matrix by a factor of 1000 and rounded to the nearest whole number. The number of subgroups was selected using 5-fold cross-validation via the *selectk()* function (*MetaTopics*). LDA models for subgroup numbers between 5 and 190 were fitted and compared based on perplexity and loglikelihood values. Both parameters continued to improve with increasing subgroup number without a clear optimum, but the first jump in model performance was seen between 20 and 25 subgroups. As a relatively small subgroup number was necessary for this analysis to allow for interpretability, this subgroup number range was chosen for further analysis. In a sensitivity analysis performed with all 2091 OTUs, a small jump was seen between 15 and 20 subgroups. Five models with *k* = 20 were then fitted and compared.

#### Diet-subgroup and disease-subgroup associations

Associations between habitual diet and microbial subgroups were assessed using Dirichlet regression models (R package *DirichletReg* version 0.7.0), which are able to evaluate associations between predictor variables and multiple compositional outcome variables [55]. This was necessary in our case, as each subgroup corresponds to one variable, and together the 20 subgroup variables are compositional (i.e., values across the subgroups add up to 1 for each observation). One model was fitted for each of 29 selected food items or nutrients and two diet quality scores with all 20 subgroups as the response variables. These models were limited to the 1442 participants for whom both nutrition and microbiota data are available. Each model was adjusted for age, sex, energy intake, education, smoking, and physical activity. All 1442 participants had complete covariate information. Estimates were given per standard deviation for each dietary factor.

The associations between selected metabolic diseases or risk factors (body mass index (BMI), waist circumference, HDL-c, LDL-c, total cholesterol, triglycerides, diabetes, hypertension) and microbial subgroups were also evaluated using Dirichlet regression models. One model was fitted per disease or risk factor (8 models). All disease models were adjusted for age, sex, education, smoking, and physical activity. The serum lipid models were additionally adjusted for use of lipid-lowering medications. For each model, any participants with missing covariate information were excluded from the analysis; for the diabetes and serum lipid models, participants who were not fasted before the blood draw were also excluded (lipids, *n* = 20; BMI/waist circumference, *n* = 2; diabetes, *n* = 16; hypertension, *n* = 3). This resulted in a sample size of *n* = 1976 for the diabetes model, *n* = 1972 for the lipid models, *n* = 1990 for the BMI and waist circumference models, and *n* = 1989 for the hypertension model. Estimates were given per standard deviation for each continuous variable (BMI, waist circumference, HDL-c, LDL-c, total cholesterol, triglycerides). For the diabetes and hypertension models, the reference categories were normal glucose tolerance and normal blood pressure, respectively. *P* values for all associations were adjusted using the Bonferroni correction (*α* = 0.05 / 39 = 0.00128).

#### Subanalysis of arrhythmic OTUs

In a previous analysis, we identified time of defecation as one of the main factors responsible for interindividual differences in microbiota composition in the KORA FF4 cohort [22]. A heat map of the normalized relative abundances of 422 OTUs clearly showed daytime-dependent fluctuations in peak relative abundance. Strikingly, a subset of 87 of these OTUs lost their daytime-dependent fluctuations in relative abundance and became arrhythmic in T2DM and/or obesity. We identified a diabetes risk signature of 13 of these OTUs that were linked to disrupted circadian rhythmicity in microbial profiles. An additional 51 OTUs were identified as losing their rhythmicity in obesity specifically. A classification model including the diabetes-specific arrhythmic OTUs was able to predict type 2 diabetes in participants 5 years after the initial sampling. This indicates that the loss of rhythmicity may play a role in the development of these disease states and contributes significantly to the classification and prediction of type 2 diabetes mellitus.

Because LDA is able to identify hidden underlying patterns in a data set, and it was previously determined that time of defecation was one of the main factors responsible for interindividual differences in microbiota composition, we would expect LDA to pick up this effect and identify subgroups of microbes which are strongly influenced by circadian rhythm. Therefore, a subanalysis was done to determine the proportion of each subgroup comprised of OTUs identified as losing their circadian rhythmicity in either obesity, T2DM, or both. For each subgroup, the probabilities for each group of arrhythmic OTUs were summed up, both for all 87 arrhythmic OTUs and for the OTUs specific to obesity and T2DM (51 and 14 OTUs, respectively).

#### Subanalysis of enterotypes

To further explore the appropriateness of LDA in comparison to more traditional methods for identifying diet- and disease-microbiota relationships, three clusters, similar to enterotypes originally identified by Arumugam et al., were identified within the data set [45]. Further details of the clustering method and characteristics of the clusters utilized in the present subanalysis are reported in [22]. Briefly, the three clusters identified, C1 (*n* = 666), C2 (*n* = 1076), and C3 (*n* = 250), were dominated by the genus *Bacteroides*, *Ruminococcus*, and *Prevotella*, respectively. In the analyses restricted to participants with dietary data (*n* = 1442), clusters 1–3 contained 473, 798, and 171 participants, respectively. The relationships between diet, metabolic diseases, and enterotypes were evaluated using multinomial logistic regression models, with C2 (*Ruminococcus*) set as the reference level. The exposure and adjustment variables were identical to those in the Dirichlet regression models and again continuous variables were divided by standard deviation. Reference categories for the diabetes and hypertension variables were “normal glucose tolerance” and “normal blood pressure,” respectively. *P* values for all associations were adjusted using the Bonferroni correction (*α* = 0.05 / 39 = 0.00128).

#### Descriptive statistics and figures

Mean and standard deviation were calculated for continuous variables, while percentage and frequency were calculated for categorical variables for the descriptive tables, for the total population and stratified by sex. The violin plot, histogram, and bar plots were generated using *ggplot2* version 3.3.1 in R. Hierarchical clustering of the subgroups was performed on log-transformed data using the *agnes*() function (*cluster* package version 2.1.0) and Ward’s method. The full matrix containing probabilities for each OTU was used for clustering to include potential differences in species, which would not be taken into account if probabilities were collapsed to the genus level. The dendrogram and cluster visualization were produced with the *factoextra* package version 1.0.7. A feature-expression heat map displaying the beta coefficients and *P* values from the Dirichlet regression models was created using the *corrplot* package version 0.84 in R and combined in Inkscape, as suggested by Haarman et al. [56]. All statistical analyses were conducted in RStudio Version 1.1.423 and R for Windows Version 3.5.1.

## Results

### Study population

The descriptive characteristics of the study population are shown in Table 1, for the total population and stratified by sex. On average, men (*n* = 969) were 61 years old and women (*n* = 1023) were 60 years old. Men had a higher mean waist circumference and BMI (103 cm and 28.3 kg/m^2^, respectively) than women (91 cm and 27.4 kg/m^2^, respectively). Women had a lower level of education on average (69.8% of women with < 13 years vs. 60.2% of men), but a higher percentage of women were physically active during leisure time (59.2% of women vs. 55.4% of men) and had never smoked (53.3% of women vs. 38.8% of men). In men, there was a 12.3% prevalence of T2DM compared to 7.9% in women (10.0% total). While this is higher than the prevalence T2DM in the general population, which was estimated to be between 6.9 and 7.1% in 2009 and 2010, respectively, this is to be expected as the average age of participants in our study population was 60.37 years, and the prevalence of T2DM rises sharply with increasing age [57].

**Table 1:** Characteristics of the study population by sex

	Total		Men		Women	
	*n* = 1992		*n* = 969		*n* = 1023	
Continuous variables	Mean	SD	Mean	SD	Mean	SD
Age (years)	60.37	12.24	60.83	12.53	59.94	11.95
Waist circumference (cm)	96.95	14.26	102.91	12.28	91.31	13.71
BMI (kg/m^2^)	27.85	5.01	28.30	4.52	27.43	5.40
HDL-c (mmol/l)	1.70	0.49	1.50	0.40	1.89	0.49
LDL-c (mmol/l)	3.48	0.92	3.45	0.90	3.51	0.94
Total cholesterol (mmol/l)	5.59	1.02	5.43	1.00	5.75	1.01
Triglycerides (mmol/l)	1.39	0.83	1.56	0.98	1.24	0.63
Categorical variables	%	*n*	%	*n*	%	*n*
Education						
< 13 years	65.1	1297	60.2	583	69.8	714
≥ 13 years	34.8	693	39.7	385	30.1	308
NA	0.1	2	0.1	1	0.1	1
Physical activity						
Active	57.4	1143	55.4	537	59.2	606
Inactive	42.6	849	44.6	432	40.8	417
Smoker						
Current	15.2	302	15.7	152	14.7	150
Ex-	38.6	769	45.5	441	32.1	328
Never	46.2	921	38.8	376	53.3	545
Diabetes						
Normal glucose tolerance	47.8	952	37.4	362	57.7	590
Prediabetes	34.2	681	42.3	410	26.5	271
UDM	4.2	84	5.2	50	3.3	34
Prevalent T2DM	10.0	200	12.3	119	7.9	81
Unknown/other	3.8	75	2.9	28	4.6	47
Hypertension						
Normal blood pressure	60.3	1202	55.3	536	65.1	666
Known HTN, controlled	26.3	523	26.6	258	35.9	265
Known HTN, uncontrolled	6.8	136	8.4	81	5.4	55
Known HTN, not treated	4.0	80	5.7	55	2.4	25
Undiagnosed HTN	2.5	50	3.9	38	1.2	12
NA	0.1	1	0.1	1	0.0	0
Lipid-lowering medication (yes)	16.5	328	19.4	188	13.7	140

*SD*, standard deviation; *BMI*, body mass index; *HDL-c*, high-density lipoprotein cholesterol; *LDL-c*, low-density lipoprotein; *UDM*, undiagnosed diabetes mellitus; *T2DM*, type 2 diabetes mellitus; *HTN*, hypertension

Hypertension was also more prevalent among men than women, particularly in the uncontrolled and untreated categories (8.4% vs. 5.4% and 5.7% vs. 2.4%, respectively).

A description of habitual dietary intake in the study population is presented in Table 2, for the total population and stratified by sex. On average, women consumed more fruits (161 g/day vs. 153 g/day), vegetables (192 g/day vs. 157 g/day), dairy products (128 g/day vs. 100 g/day), and yogurt (53.6 g/day vs. 40.0 g/day), whereas men had a notably higher energy intake (2127 kcal/day vs. 1619 kcal/day) and consumption of refined grains (170 g/day vs. 121 g/day), red and processed meat (56.0 g/day and 65.6 g/day respectively in men vs. 35.4 g/day and 34.5 g/day respectively in women), sugar and sweets (39.7 g/day vs. 15.8 g/day), sugar-sweetened beverages (SSB, 77.4 g/day vs. 30.6 g/day), beer (274 g/day vs. 21.9 g/day), and total alcohol (15.9 g/day vs. 4.5 g/day). Women had a higher AHEI score than men (45.6 vs. 40.7), but scored slightly lower on the MDS (4.3 vs. 4.6).

**Table 2:** Habitual dietary intake of the study population by sex

	Total		Men		Women	
	*n* = 1442		*n* = 699		*n* = 743	
	Mean	SD	Mean	SD	Mean	SD
Food items						
Potatoes (g/day)	60.91	22.45	66.57	23.17	55.59	20.38
Vegetables (g/day)	174.66	57.78	157.03	47.36	191.24	61.68
Legumes (g/day)	6.13	4.43	5.41	3.89	6.82	4.79
Fruit (g/day)	156.86	80.16	152.71	80.75	160.77	79.45
Nuts and seeds (g/day)	7.54	8.09	8.16	8.66	6.95	7.47
Dairy products (g/day)	114.48	86.27	100.05	82.70	128.06	87.40
Yogurt (g/day)	47.00	45.56	39.97	43.26	53.62	46.69
Cheese (g/day)	35.00	18.47	36.63	19.07	34.40	17.89
Refined grains (g/day)	144.52	41.80	169.60	37.85	120.94	29.94
Whole grains (g/day)	23.50	20.63	24.05	22.90	22.97	18.23
Fresh red meat (g/day)	45.41	15.92	56.02	15.03	35.42	8.57
Processed (red) meat (g/day)	49.60	28.21	65.61	29.83	34.54	15.46
Fish and shellfish (g/day)	21.03	13.82	23.63	16.13	18.59	10.68
Eggs (g/day)	17.23	11.56	18.38	12.77	16.15	10.19
Animal fats (g/day)	15.92	6.75	18.73	7.32	13.28	4.87
Plant oils (g/day)	9.09	6.71	10.58	8.23	7.69	4.42
Sugar and sweets (g/day)	37.63	15.27	39.74	35.64	15.77	14.51
Cakes (g/day)	53.85	19.83	58.57	21.52	49.41	16.94
Coffee (g/day)	401.48	133.81	408.95	141.87	394.46	125.45
SSB (g/day)	53.28	132.34	77.40	168.77	30.59	78.45
Wine (g/day)	38.07	51.59	43.24	58.15	33.21	44.03
Beer (g/day)	144.30	208.56	274.39	233.58	21.92	47.24
Nutrients						
Energy intake (kcal/day)	1865.35	405.36	2127	350	1619	281
Total fat (g/day)	78.08	17.06	88.41	15.52	68.37	12.00
Total carbohydrates (g/day)	199.66	49.43	223.27	48.31	177.45	39.11
Total protein (g/day)	69.89	14.85	78.31	13.73	61.97	11.00
Total fiber (g/day)	17.85	5.05	18.43	5.14	17.31	4.90
Soluble fiber (g/day)	5.79	1.60	6.11	1.64	5.49	1.51
Insoluble fiber (g/day)	11.96	3.44	12.29	3.48	11.65	3.37
Alcohol (g/day)	10.03	10.07	15.89	10.96	4.51	4.59
Dietary patterns						
Alternate Healthy Eating Index	43.23	9.27	40.71	8.75	45.61	9.11
Mediterranean Diet Score	4.43	1.68	4.59	1.45	4.28	1.86

*SD*, standard deviation; *SSB*, sugar-sweetened beverages

#### Composition of the subgroups

**Fig. 1:**
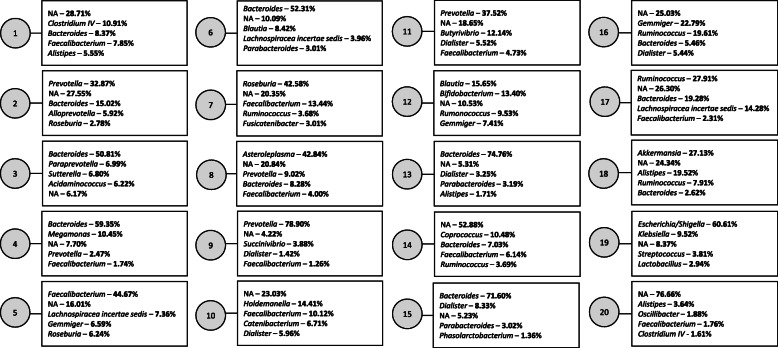
The top five genera (ranked by probability) per subgroup. For each subgroup, the five genera with the highest probability per subgroup are displayed. All OTUs for which the genus was unknown were grouped into an NA category; the percentage corresponding to NA indicates the percentage of the subgroup comprised of OTUs which could not be identified to the genus level

**Fig. 2:**
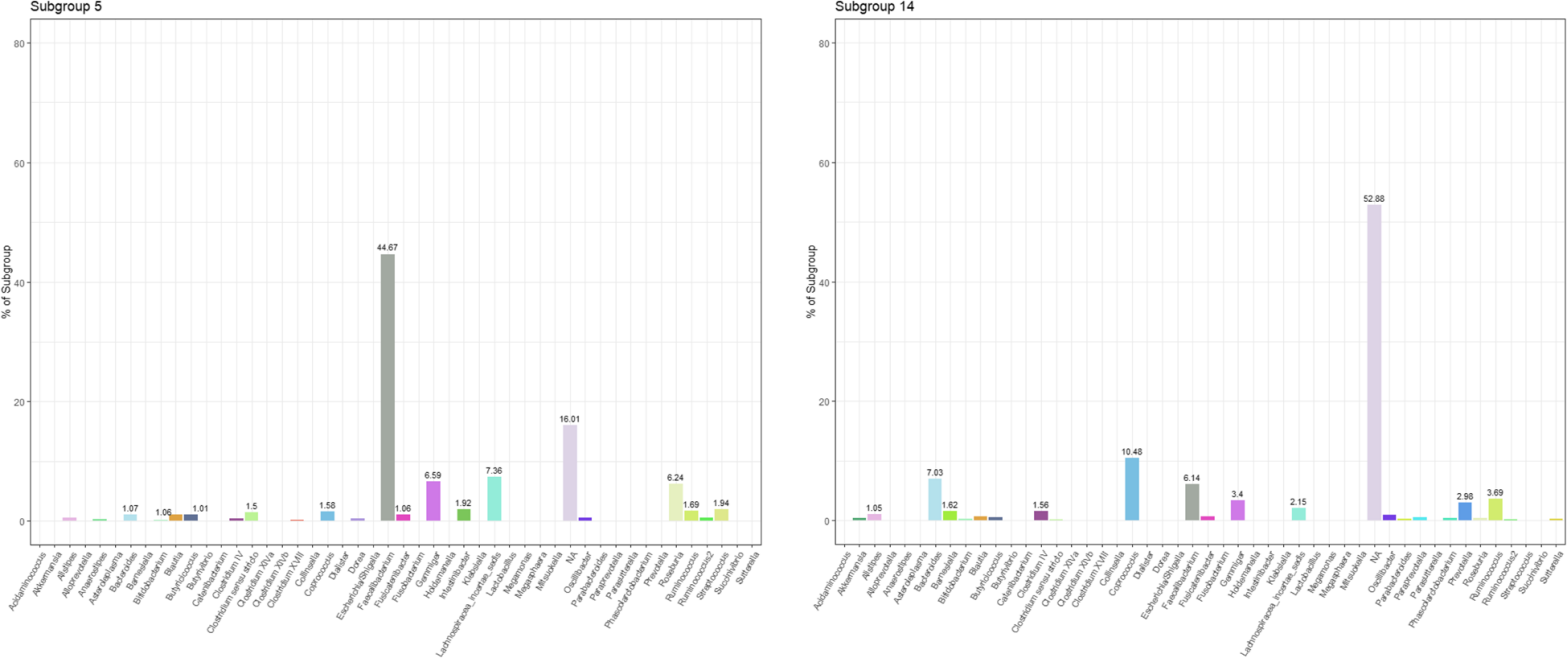
All 47 (of 97 total) genera that contributed to > 1% of any subgroup are shown, plus one NA category which encompasses all OTUs for which the genus was unknown

**Fig. 3:**
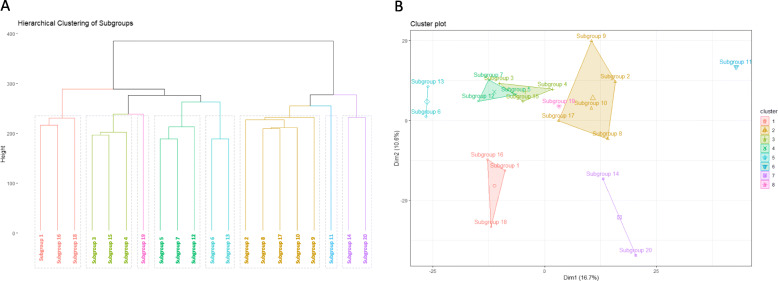
Hierarchical clustering of subgroups. **a** Dendrogram of subgroup clusters for comparison of subgroup similarity. Eight clusters are displayed, each represented by a different color and outlined by a gray rectangle. **b** Visualization of subgroup clusters. Cluster colors correspond to cluster colors from **a**

Figure 1 displays the top five genera for each of the 20 subgroups identified in the study population. The OTU probabilities were collapsed to the genus level here to allow for better interpretation of subgroup composition. Subgroup numbering is random and serves only as an identifier. While some subgroups (e.g., subgroups 1, 10, 11, 12, 14) were comprised of several different genera, others (e.g., subgroups 3, 4, 9, 13, 15, 19) were composed of more than 50% from a single genus. Figure 2 displays a visual comparison of the composition of subgroups 5 and 14, the two subgroups most strongly associated with both diet and disease, by genus. Only the 47 genera representing at least 1% of any subgroup plus are displayed, plus an NA category that encompassed all OTUs for which the genus was unknown. Some of the main genera in these two subgroups overlap (*Faecalibacterium*, *Bacteroides*, *Coprococcus*, *Roseburia*), though they are present in very different proportions in each subgroup. Additionally, some genera are prevalent in one subgroup but not the other (e.g., *Intestinibacter* and *Streptococcus* in subgroup 5, *Barnesiella* and *Prevotella* in subgroup 14). Notably, subgroup 14 was comprised 52.88% of OTUs that could not be identified to the genus level. Additional file 1 details the full composition of each subgroup by genus (97 genera in total were identified plus one overarching NA category). Figure 3 displays the results of hierarchical clustering of the subgroups. The clustering was performed on a matrix containing the probabilities of each of the 1713 unlabeled OTUs for each of the twenty subgroups. Eight clusters are each highlighted with a unique color to aid in comparison of subgroup similarity. Interestingly, although subgroups 3, 4, 6, 13, and 15 were all dominated by *Bacteroides*, they are split into two different clusters, which may be a result of different *Bacteroides* species which tend to appear in subgroups from one cluster vs. the other. Furthermore, although subgroups 13 and 15 appear very similar in regard to the composition of their top five genera, they also belong to different clusters. Additionally, though subgroups 5 and 14 appeared to have vaguely similar taxonomic compositions in Fig. 2, they were also assigned to different clusters, potentially also due to OTUs that differ at the species level. This highlights the importance of microbiota data that can reliably be identified beyond the genus level, but also the strength of LDA in identifying OTUs from a single genus that may represent different species and may, as a result, have differing functions or characteristics.

**Fig. 4:**
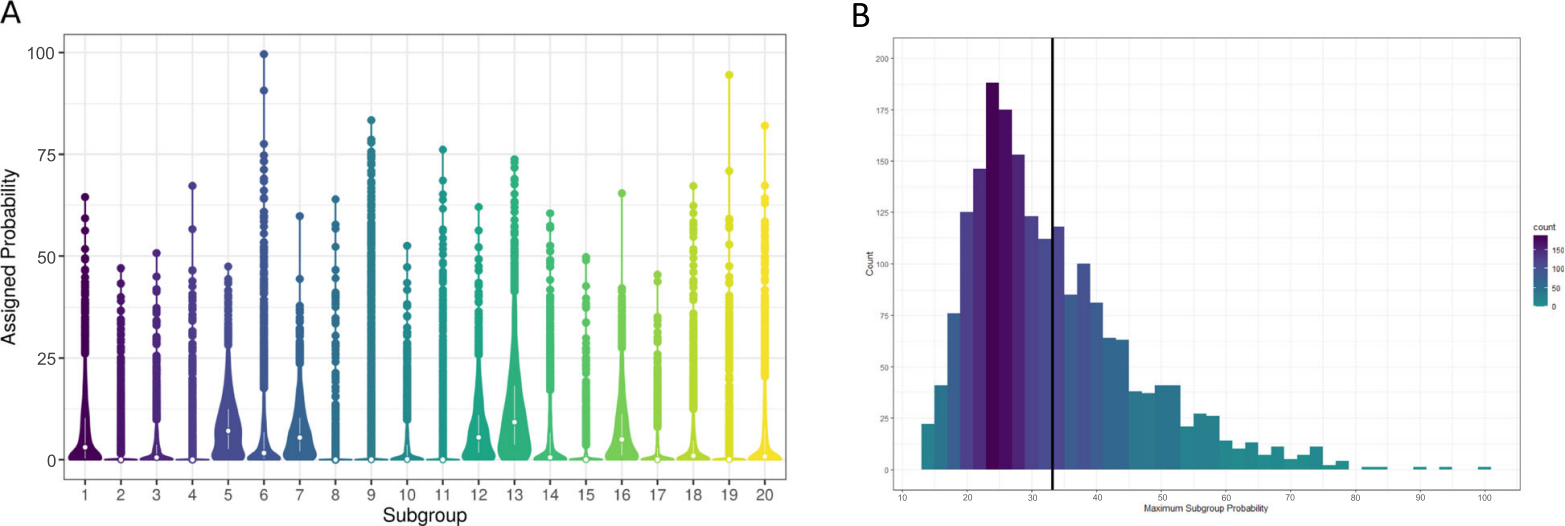
Distribution of subgroups across the study population. **a** Violin plot showing the probability per subgroup among the study population. Each violin displays the distribution of that subgroup across all participants. The median and interquartile range for each subgroup is represented by a white dot and bar. **b** Histogram showing the maximum probability for any subgroup per participant. The mean maximum probability was 33.24% ± 1.27% (indicated by vertical line). Each bin represents the participant count for that probability range. The darker the bin color, the higher the count

Figure 4a describes the distribution of subgroups among the study population. Some subgroups were quite prevalent, with a median probability across the study population of between 3.1 and 9.2% (subgroups 1, 5, 7, 12, 13, and 16), while others were rarer, with a median probability of < 0.1% (subgroups 2, 4, 8, 9, 10, 11, 15, 17, 19). Figure 4b shows the maximum subgroup probability across participants. Participants had an average maximum subgroup probability of 33.2%, although the highest maximum probability of any individual for any subgroup was 99.6%, and the lowest maximum probability of any individual was 13.1% (Fig. 4b). On average, each participant had 10 ± 2.2 subgroups with a probability of over 1%, 4.5 ± 1.1 subgroups with a probability of over 10%, and 1.5 ± 1.1 subgroups with a probability greater than 25%.

#### Habitual diet and subgroups

**Fig. 5:**
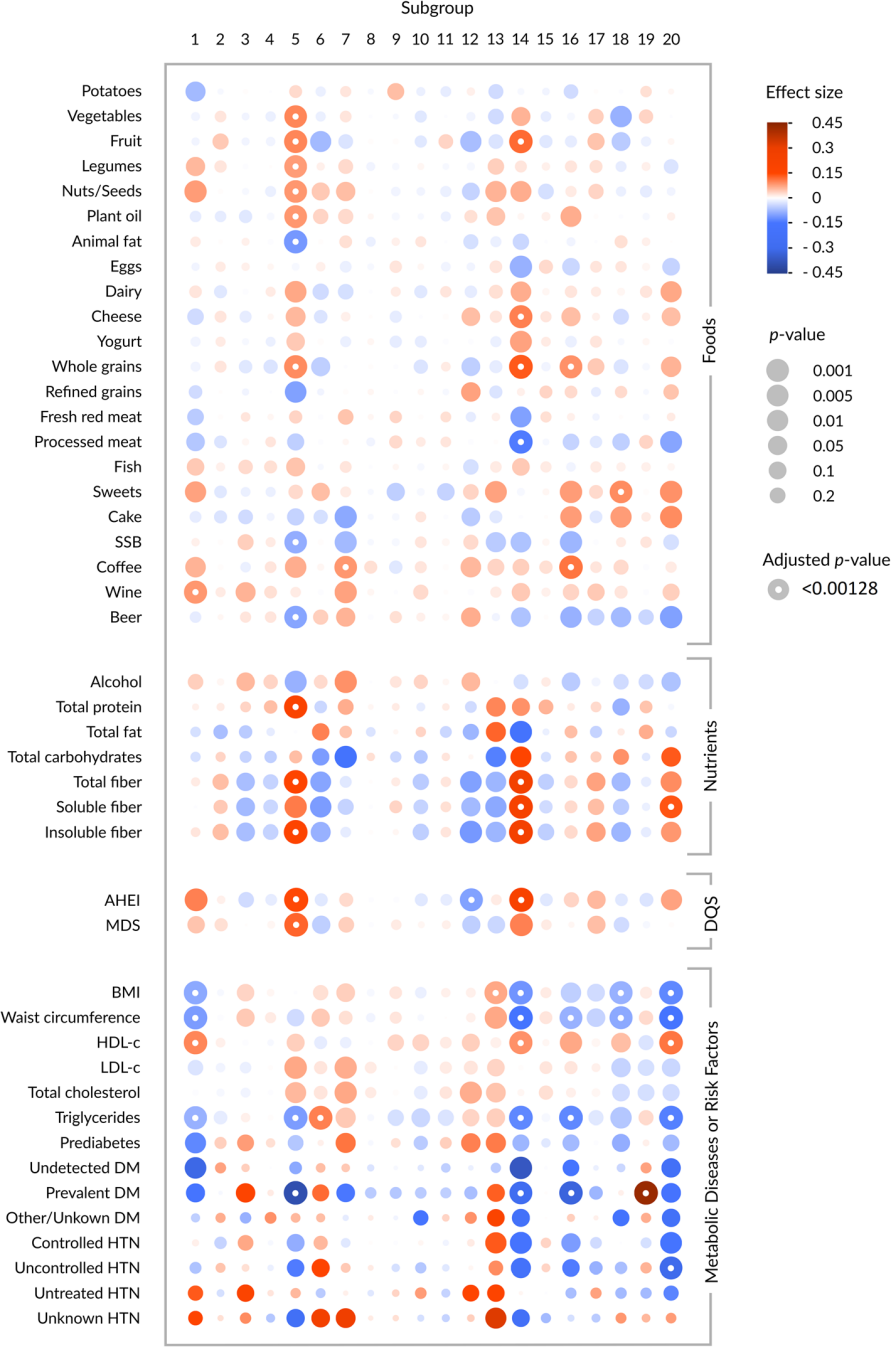
Feature expression heat map displaying results of the Dirichlet regression models. *SSB*, sugar-sweetened beverages; *AHEI*, Alternate Healthy Eating Index; *MDS*, Mediterranean Diet Score; *DM*, diabetes mellitus; *HTN*, hypertension; *DQS*, diet quality score. Foods, nutrients, or DQS and subgroups were analyzed in one set of models, while metabolic diseases or risk factors and subgroups were analyzed in another. Each association between a dietary factor or metabolic disease/risk factor and a subgroup (as identified in the Dirichlet regression models) is represented by a circle, where the size of the circle indicates the significance of the association (size inversely proportionate to *P* value), and intensity of the color (red, positive; blue, inverse) indicates the effect size. A white dot in the center of a circle indicates that the association remained significant after Bonferroni correction (*P* < 0.00128)

The associations between habitual diet (22 food items, seven nutrients, and two diet quality scores) and the 20 subgroups are displayed in Fig. 5. Subgroups 2, 4, 8, 9, 10, 11, 15, and 19 were not associated with any dietary factors. After adjustment for multiple testing, associations between diet and subgroups 1, 5, 7, 12, 14, 16, 18, and 20 remained significant. Subgroups 5 and 14 were most strongly and consistently associated with several nutrition factors. Participants with a high probability for subgroup 5 were characterized by a high intake of vegetables, fruits, legumes, nuts and seeds, plant oils, whole grains, total protein, total fiber, and insoluble fiber, and a low consumption of animal fat, SSB, and beer. A higher probability for subgroup 5 was also associated with a higher MDS and AHEI score. Those with a high probability for subgroup 14 were characterized by a higher AHEI score, as well as high consumption of fruit, cheese, whole grains, and all types of fiber, and a low consumption of processed meat. Individuals with a higher probability for subgroup 16 were marked by greater consumption of whole grains and coffee, while a higher probability for subgroup 20 was significantly associated with higher soluble fiber intake. Uniquely, subgroup 12 was significantly inversely associated with the AHEI, but not with any individual food item or the MDS after adjustment with the Bonferroni correction. Interestingly, many of the associations that lost significance after adjustment for multiple testing were with dietary factors that tend to be associated with adverse health effects, whereas many of the diet-subgroup associations that were the strongest were with dietary factors considered to be health-promoting. The full results of the Dirichlet regressions between habitual diet and the subgroups are shown in Additional file 2.

#### Metabolic diseases or risk factors and subgroups

The associations between selected metabolic diseases or risk factors and the 20 subgroups are also presented in Fig. 5 (full results in Additional file 3). Subgroups 1, 3, 5, 6, 7, 12, 13, 14, 16, 18, 19, and 20 were initially associated with one or more metabolic diseases or risk factors. After adjustment for multiple testing, only significant associations with subgroups 1, 5, 6, 13, 14, 16, 19, and 20 remained. The subgroups that showed the strongest/most numerous associations with diet (subgroup 5 (*Faecalibacterium*, *Lachnospiracea incertae sedis*, *Gemmiger*, *Roseburia*), subgroup 14 (*Coprococcus*, *Bacteroides*, *Faecalibacterium*, *Ruminococcus*), and, to a lesser extent, subgroup 16 (*Gemmiger*, *Ruminococcus*, *Bacteroides*, *Dialister*)) also showed strong associations with metabolic diseases or risk factors. Participants with lower serum triglyceride levels and a low prevalence of T2DM showed a higher probability for any of these three subgroups. Individuals with a lower BMI and/or waist circumference had a higher probability for subgroups 14 or 16, while higher serum HDL-c levels were associated with a higher percentage of subgroup 14.

**Fig. 6:**
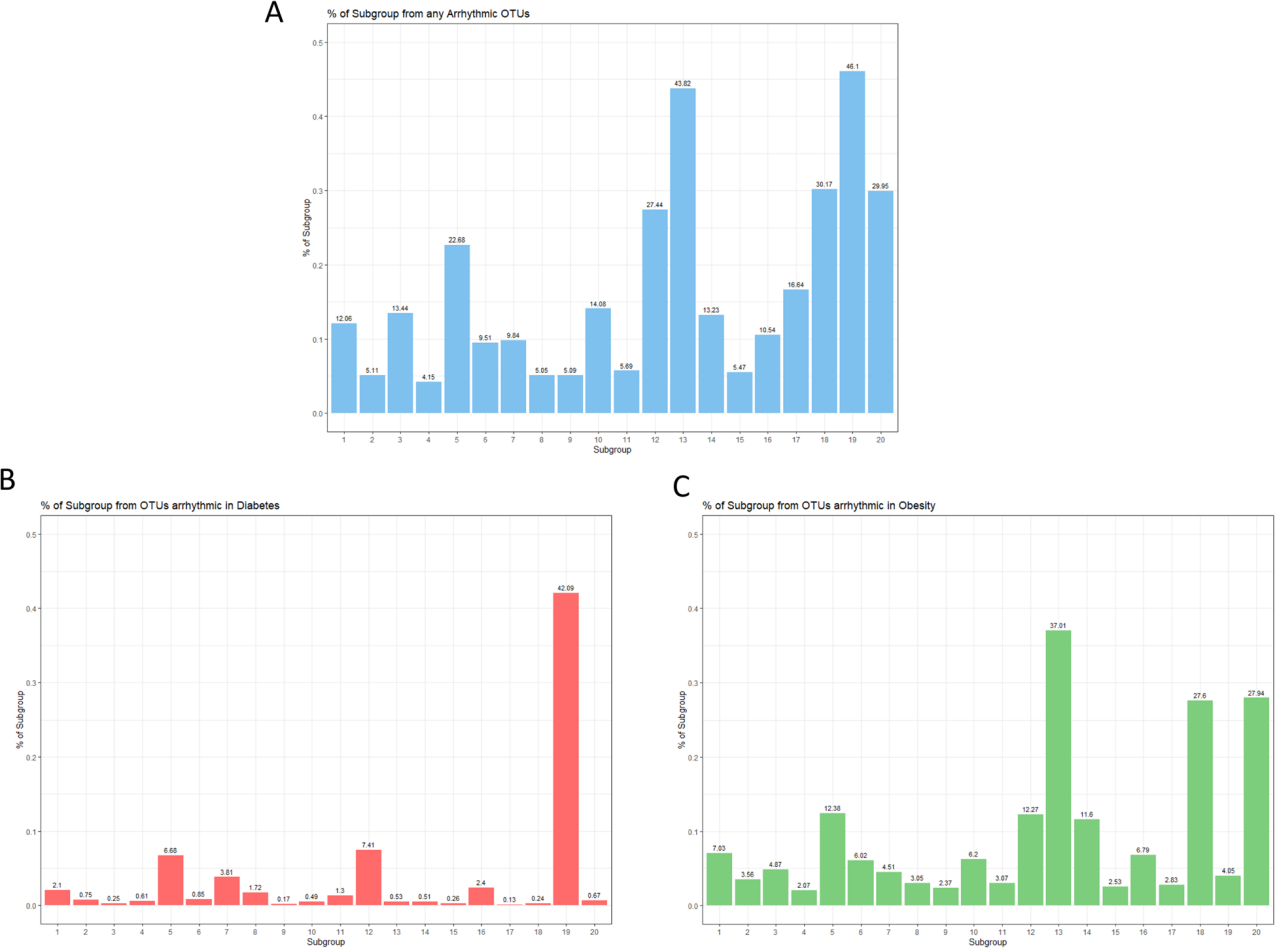
Percentage of each subgroup from arrhythmic OTUs. **a** Percentage of each subgroup from any arrhythmic OTUs. **b** Percentage of each subgroup from diabetes-specific arrhythmic OTUs. **c** Percentage of each subgroup from obesity-specific arrhythmic OTUs

As a continuation of our previous analysis, in which we identified 87 OTUs that normally show daytime-dependent fluctuations in peak relative abundance but lose their rhythmicity in T2DM and/or obesity, we calculated the distribution of these arrhythmic OTUs among the subgroups (Fig. 6) [22]. Subgroups 19 (*Escherichia*/*Shigella*, *Klebsiella*, *Streptococcus*, *Lactobacillus*) and 13 (*Bacteroides*, *Dialister*, *Parabacteroides*, *Alistipes*) had a much higher percentage of arrhythmic OTUs than the rest (46% and 44%, respectively), followed by subgroups 18, 20, 12, and 5 (30%, 30%, 27%, and 23% arrhythmic OTUs, respectively). The remaining subgroups ranged between 5 and 17% arrhythmic OTUs. Regarding the 14 OTUs that were found to be arrhythmic in T2DM specifically, subgroup 19 had a notably higher proportion, with 42% of the subgroup being composed of diabetes-specific arrhythmic OTUs (all other subgroups contained only between 0 and 7%). Regarding the 51 OTUs found to be obesity-specific, subgroups 13, 18, and 20 had notably high percentages (37%, 28%, and 28%, respectively; the remaining subgroups ranged from 2 to 12%).

Additionally, we conducted a subanalysis of the associations between habitual diet, metabolic diseases/risk factors, and three enterotype-like clusters. After correction for multiple testing, no dietary factors were significantly associated with enterotype (Additional file 4). BMI and waist circumference were positively associated with the *Bacteroides* cluster (OR (CI): 1.34 (1.21–1.49); 1.43 (1.27–1.61) respectively), as well as serum triglycerides (1.29 (1.16–1.43)) and undetected and prevalent diabetes (2.45 (1.47–4.07), 1.85 (1.28–2.67), respectively). Waist circumference was positively associated with the *Prevotella* enterotype (OR (CI): 1.31 (1.11–1.54)).

## Discussion

We used LDA to identify 20 latent microbial subgroups in human gut microbiota samples from 1992 participants of the KORA FF4 study. We chose this method because of its wide use in other fields and its unique applicability to microbiota data [47]. Unlike clustering, LDA is a generative machine learning model, which is able to detect latent, or hidden, groups within data. Rather than using distance between observations as a measure, it identifies patterns of co-occurrence. As a result, each subgroup represents microbes that typically appear together in an environment (sample), likely due to similar environmental requirements (e.g., similar nutrient/substrate needs), similar roles or functions within an environment, or because the taxa within a subgroup are modulated by a shared, latent factor (e.g., medication, circadian rhythm, or other potentially unknown factors). The factors driving the co-occurrence of taxa within a subgroup may or may not be clear from a priori knowledge. As a result, LDA offers a unique advantage over traditional methods of clustering or drawing associations with individual taxa, as it can identify patterns within a data set that are driven by latent factors that researchers may or may not already be aware of and which can inherently not be detected by other methods of analysis. Additionally, traditional clustering methods are strongly influenced by dominant taxa, and are likely to miss more obscure features, which may be just as relevant. A further advantage of this strategy is that it is not limited to identifying individual taxa which may be beneficial or harmful, but rather a group of taxa that may act synergistically, have similar beneficial roles, produce similar metabolites, etc. Finally, LDA allows for partial membership, meaning each sample may contain several microbial subgroups, which is more appropriate to the biological situation and allows for a more complex description of the microbial environment than hard clustering methods. These unique advantages of LDA could prove useful for informing future studies by identifying subgroups of microbes within a population that are relevant for human health and disease, and which may be modulated by hidden factors that warrant further investigation. Several recent studies have demonstrated the advantages of LDA for metagenomic data. One excellent example of this is a recent analysis by Hosoda et al., in which LDA was utilized to identify four microbial “assemblages” (subgroups) within a metagenomic data set [50]. Three of these assemblages correlated strongly with the classic three-cluster enterotypes, but the fourth assemblage, which was present within each of the three enterotypes, was not as dominant within the data as the three more distinct assemblages and has therefore missed by classic analysis methods in the past.

Another paper, by authors Sankaran and Holmes, demonstrated the applicability of LDA to microbiome data using both a case study of the effect of two antibiotic courses on microbiota composition and a simulated microbiome data set [47]. The authors demonstrated that LDA was capable of appropriately modeling the simulation data set and was also able to identify four microbial “topics” (subgroups) in the data set, each of which responded uniquely to the antibiotic courses over time, despite a lack of temporal information.

In the present analysis, using multivariate Dirichlet regression models, one set that examined diet and subgroups, and another set of models that examined diseases/risk factors and subgroups, we identified a number of significant associations between habitual diet or metabolic diseases/risk factors and microbial subgroups. Subgroups 5 and 14 were most strongly and consistently associated with dietary factors and at the same time with metabolic diseases or risk factors. Many of these associations are consistent with current knowledge on diet-metabolic disease associations that were newly attributed to specific microbial subgroups in our analysis. This gives sound support to the hypothesis that diet-disease associations are at least partially modulated by the subject’s microbiota structure, as reflected by these two main subgroups—though causality remains to be confirmed.

The dietary associations we identified with subgroup 5 (*Faecalibacterium*, *Lachnospiracea incertae sedis*, *Gemmiger*, *Roseburia*) are consistent with that of a diet protective against T2DM. Interestingly, one study found that adhering to a Mediterranean diet increased occurrence of *Faecalibacterium prausnitzii* in feces and improved insulin sensitivity [58, 59]. This is consistent with our findings that closer adherence to a Mediterranean diet was associated with higher probability of subgroup 5 (44.67% *Faecalibacterium*; inversely associated with prevalent T2DM). Furthermore, several additional studies have shown an increase in *F. prausnitzii*, often as a result of modulation with a high-fiber diet, to be associated with improvement in T2DM parameters [19, 60, 61]. Subgroup 5 has the highest percentage of *Faecalibacterium*, but it is among the top five genera in other subgroups as well (e.g., subgroups 7, 10, 11). However, most of these subgroups showed no association with diet or disease markers, indicating the value of looking at a group of co-occurring bacteria rather than individual taxa.

Subgroup 14 (*Coprococcus*, *Bacteroides*, *Faecalibacterium*, *Ruminococcus*) was significantly positively associated with fruit, cheese, whole grains, and total, soluble and insoluble fiber intake after adjustment with the Bonferroni correction, and inversely with processed meat. These associations with subgroup 14 are logical, as *Coprococcus* species are fiber-fermenting butyrate producers, and their presence has generally been associated with positive health states [62–66].

Soluble fiber was the only dietary factor to remain associated with subgroup 20 after adjustment with the Bonferroni correction, although subgroup 20 was significantly associated with BMI, waist circumference, and several other metabolic parameters. Subgroup 20 was also one of the subgroups with a high percentage of arrhythmic OTUs; 28% of the subgroup was comprised of OTUs identified as behaving arrhythmically in obesity (Fig. 6). These findings support the associations we identified between subgroup 20 and BMI and waist circumference. Unfortunately, subgroup 20 is comprised of over 75% of OTUs that could not be reliably identified to the genus level, although its strong associations with disease and high percentage of OTUs that are arrhythmic in obesity suggest this subgroup could be of great interest for further research.

Subgroup 13 (*Bacteroides*, *Dialister*, *Parabacteroides*, *Alistipes*) comprised 74.76% of the genus *Bacteroides* and was significantly positively associated with BMI. After adjustment for multiple testing, subgroup 13 was no longer significantly associated with any food item or nutrient, which suggests that another factor may be its main driver. Interestingly, 37% of subgroup 13 is composed of OTUs that were identified in our previous analysis as losing their rhythmicity in obesity (Fig. 6) [22]. Because subgroup 13 is so strongly associated with BMI, it warrants further investigation. The high percentage of obesity-specific arrhythmic OTUs in this subgroup indicates that it may be worth further investigation regarding the importance of arrhythmic OTUs and obesity in general.

Subgroup 18 (*Akkermansia*, *Alistipes*, *Ruminococcus*, *Bacteroides*) was significantly inversely associated with BMI and waist circumference. Numerous studies have previously reported inverse associations between *Akkermansia* (27.13% of subgroup 18) and obesity, which is consistent with our results [67, 68]. Conversely, the genus *Alistipes* (19.52% of subgroup 18) has been associated with both positive and negative health states, potentially depending on the host environment. Our results suggest a potentially protective effect against obesity when co-occurring with other taxa in subgroup 18 in this study population. Additionally, 30% of subgroup 18 was comprised of OTUs previously identified as becoming arrhythmic in obesity (Fig. 6), again highlighting this subgroup as potentially highly relevant to obesity.

Subgroup 19 (*Escherichia*/*Shigella*, *Klebsiella*, *Streptococcus*, *Lactobacillus*) was not associated with any dietary factor. However, there was a significant positive association between prevalent T2DM and subgroup 19. It is possible that this association is driven by the intake of the drug metformin by participants with prevalent T2DM. Indeed, one previous study found an association between the severity of gastrointestinal side effects and the relative abundance of *Escherichia-Shigella* in participants receiving metformin after just 24 h [69], and several other studies have found an increase in *Escherichia* or *E. coli* in participants taking metformin [13, 70]. Additionally, 46% of subgroup 19 was comprised of arrhythmic OTUs, 91% of which were OTUs identified in our previous analysis as part of a diabetes risk signature [22]. This is a striking difference to the other subgroups, of which only 0–7% were composed of these diabetes-specific arrhythmic OTUs. The strong association of subgroup 19 with T2DM and its large percentage of diabetes-specific arrhythmic OTUs demonstrates how effectively LDA recognized a subgroup of OTUs previously demonstrated as specific to T2DM and which lose their rhythmicity in T2DM specifically. Other prominent OTUs in this subgroup should be investigated for their potential importance in T2DM as well.

Several additional subgroups were initially associated with one or more nutrition items/factors, but were no longer significant after adjustment for multiple testing (subgroups 3, 6, 13, 17). Additionally, many of the strongest diet-subgroup associations were with “healthy” dietary factors, such as whole grains and fruit, rather than those considered to have negative health effects, such as sweets, alcohol, and SSB. This indicates that the consumption of the food groups that remained statistically significant after correction for multiple testing may have a stronger impact on microbial subgroups than the ones that did not remain significant. In other words, an increase in consumption of healthy dietary constituents seems to be a more effective measure to modify gut microbiota composition (whereas the change in composition resulting from an increase in unhealthy foods is a more passive consequence.) Thus, reproduction of these findings in an independent study is urgently needed.

Several subgroups were not associated with any dietary factors (subgroups 2, 4, 8, 9, 10, 11, 15, 19) and, with the exception of subgroup 19, were also not associated with any of the covariates selected for adjustment. These independent subgroups are consistent with the idea that we still do not fully understand the factors responsible for shaping gut microbiome composition. Further analyses should seek to identify what latent factors may be driving these subgroups.

It is highly useful to be able to classify an individual’s highly complex microbiota structure into a manageable number of subgroups that can convey information about health and disease risk. The analysis of diet/metabolic disease and enterotype-like clusters demonstrated that, although habitual diet has been associated with enterotypes in the past, this three-cluster solution is not appropriate for identifying associations between habitual diet and microbiota structure in this study population [71]. While a few significant associations were seen between BMI or waist circumference, triglycerides, and T2DM and the clusters, no significant associations between diet and any enterotype cluster were identified after adjustment for multiple testing. The limitations of this clustering strategy have been discussed previously, and our results also indicate the need for a more sensitive and detailed approach to microbiota analysis [72, 73]. One major advantage that LDA offers over traditional clustering strategies is partial or fractional membership (similar to fuzzy clustering). A major criticism of enterotypes is the assignment of an individual into one cluster, ignoring often gradient-like differences in microbiota structure. Methods allowing for partial membership, such as LDA, enable an individual’s microbiota composition to be described by a combination of several microbial groups in varying proportions, which is more likely to reflect the actual complexity of an individual’s microbiome appropriately. Our results suggest that LDA, specifically, offers an appropriate alternative for the identification of latent structures within the microbiome that are of relevance to human health and disease. This method offers a practical way to characterize an individual’s microbiota and potentially to decipher more information about an individual’s health state and potential disease risk. However, the most appropriate use of this method may be in exploratory analyses, with the purpose of identifying subgroups of bacteria within a population that are driven by latent factors, and which should be investigated with further study.

Our analysis has several additional strengths, including high-quality nutrition data that characterizes habitual dietary intake, use of a large, originally population-based cohort with the opportunity for future longitudinal analyses, and the implementation of a popular, sophisticated unsupervised machine learning algorithm. Limitations include that only one stool sample was available per individual. Despite the use of a refined nutrition assessment method, measurement error is likely to persist to some degree. Due to the cross-sectional nature of this analysis, the causality of the associations cannot be determined. Generalizability of these results cannot be assumed without reproducing this analysis in other study populations and/or follow-ups of the present study population.

## Conclusions

We described 20 microbial subgroups using an unsupervised machine learning model, several of which were associated with both habitual dietary intake and metabolic disease or relevant risk factors. The diet-microbiota and disease-microbiota associations identified in this analysis add insight to the complex relationship between diet, the human microbiome, and disease. Further analyses implementing LDA in other populations and in longitudinal studies are necessary to investigate the reproducibility of the present findings.

## Supplementary Information

**Additional file 1.**

**Additional file 2.**

**Additional file 3.**

**Additional file 4.**

**Additional file 5.**

## Data Availability

The dataset analyzed during the current study is not publicly available due to restrictions imposed by the Ethics Committee of the Bavarian Medical Association to protect the privacy of the study participants. However, a request for use of the data can be made via a project agreement through the KORA.PASST platform (https://www.helmholtz-muenchen.de/en/kora/for-scientists/cooperation-with-kora/index.html). The code used in this analysis can be found in Additional file 5.

## Abbreviations

24HFL: 24-h food list
AHEI: Alternate Healthy Eating Index
BMI: Body mass index
DNA: Deoxyribonucleic acid
EPIC: European Prospective Investigation into Cancer and Nutrition
FFQ: Food Frequency Questionnaire
HDL-c: High-density lipoprotein cholesterol
KORA: Cooperative Health Research in the Region of Augsburg
LDA: Latent Dirichlet allocation
LDL-C: Low-density lipoprotein cholesterol
MDS: Mediterranean Diet Score
OTU: Operational taxonomic unit
PCR: Polymerase chain reaction
rRNA: Ribosomal ribonucleic acid
T2DM: Type 2 diabetes mellitus
