# Supplementary material for: Associations between habitual diet, metabolic disease, and the gut microbiota using latent Dirichlet allocation
Source: Microbiome. 2021 Mar 16;9:61. doi: 10.1186/s40168-020-00969-9 (PMC7967986; doi:10.1186/s40168-020-00969-9)
Supplement: Supplementary file 3 — Additional file 2. [file 40168_2020_969_MOESM3_ESM.docx]

**Additional Table 2.** Associations between dietary factors (per SD) and microbial subgroups in the gut

|  | Subgroup 1 | | | Subgroup 2 | | | Subgroup 3 | | |
| --- | --- | --- | --- | --- | --- | --- | --- | --- | --- |
| Food Item | **Est** | **SE** | ***p*** | **Est** | **SE** | ***p*** | **Est** | **SE** | ***p*** |
| Potatoes | **-0.07** | **0.03** | **0.03** | -0.01 | 0.03 | 0.87 | 0.003 | 0.03 | 0.93 |
| Vegetables | -0.01 | 0.03 | 0.69 | 0.02 | 0.03 | 0.49 | -0.01 | 0.03 | 0.77 |
| Legumes | **0.05** | **0.03** | **0.047** | 0.02 | 0.03 | 0.44 | -0.004 | 0.03 | 0.90 |
| Fruit | -0.01 | 0.03 | 0.74 | 0.04 | 0.03 | 0.18 | -0.01 | 0.03 | 0.75 |
| Nuts and seeds | **0.08** | **0.03** | **0.002** | 0.003 | 0.03 | 0.93 | -0.01 | 0.03 | 0.82 |
| Dairy products | 0.02 | 0.03 | 0.41 | -0.03 | 0.03 | 0.38 | -0.004 | 0.03 | 0.89 |
| Yogurt | -0.01 | 0.03 | 0.60 | 0.01 | 0.03 | 0.61 | -0.01 | 0.03 | 0.82 |
| Cheese | -0.04 | 0.03 | 0.16 | 0.02 | 0.03 | 0.47 | -0.02 | 0.03 | 0.45 |
| (Refined) Grains | -0.04 | 0.04 | 0.34 | 0.001 | 0.04 | 0.97 | -0.01 | 0.04 | 0.81 |
| Whole grains | -0.01 | 0.03 | 0.61 | 0.02 | 0.03 | 0.47 | -0.03 | 0.03 | 0.35 |
| Fresh red meat | -0.06 | 0.04 | 0.15 | 0.004 | 0.04 | 0.92 | 0.02 | 0.04 | 0.64 |
| Processed (red) meat | -0.06 | 0.03 | 0.07 | -0.03 | 0.03 | 0.40 | 0.01 | 0.03 | 0.81 |
| Fish and shellfish | 0.04 | 0.03 | 0.11 | 0.01 | 0.03 | 0.68 | 0.03 | 0.03 | 0.28 |
| Eggs | -0.01 | 0.03 | 0.74 | 0.01 | 0.03 | 0.59 | 0.01 | 0.03 | 0.73 |
| Animal fats | 0.02 | 0.03 | 0.58 | 0.002 | 0.03 | 0.95 | 0.01 | 0.03 | 0.75 |
| Plant oils | -0.02 | 0.03 | 0.50 | -0.02 | 0.03 | 0.50 | -0.03 | 0.03 | 0.37 |
| Sugar and sweets | **0.07** | **0.03** | **0.01** | -0.03 | 0.03 | 0.41 | -0.01 | 0.03 | 0.63 |
| Cakes | -0.02 | 0.03 | 0.48 | -0.03 | 0.03 | 0.38 | -0.04 | 0.03 | 0.26 |
| Coffee | **0.06** | **0.03** | **0.019** | 0.004 | 0.03 | 0.89 | -0.01 | 0.03 | 0.57 |
| SSB | 0.01 | 0.03 | 0.73 | 0.003 | 0.03 | 0.91 | 0.04 | 0.03 | 0.19 |
| Wine | **0.08** | **0.02** | **0.0002*** | 0.01 | 0.03 | 0.61 | **0.06** | **0.03** | **0.028** |
| Beer | -0.02 | 0.03 | 0.50 | 0.005 | 0.03 | 0.88 | 0.02 | 0.03 | 0.52 |
| Nutrient | **Est** | **SE** | ***p*** | **Est** | **SE** | ***p*** | **Est** | **SE** | ***p*** |
| Total fat | -0.03 | 0.06 | 0.57 | -0.07 | 0.06 | 0.28 | -0.06 | 0.06 | 0.36 |
| Total carbohydrates | -0.03 | 0.06 | 0.59 | 0.03 | 0.07 | 0.60 | -0.05 | 0.06 | 0.47 |
| Total protein | 0.01 | 0.05 | 0.82 | 0.02 | 0.05 | 0.75 | 0.03 | 0.05 | 0.55 |
| Total fiber | 0.02 | 0.04 | 0.68 | 0.05 | 0.04 | 0.18 | -0.07 | 0.04 | 0.07 |
| Soluble fiber | -0.001 | 0.04 | 0.98 | 0.04 | 0.04 | 0.31 | -0.07 | 0.04 | 0.06 |
| Insoluble fiber | 0.02 | 0.04 | 0.63 | 0.05 | 0.04 | 0.18 | -0.07 | 0.04 | 0.07 |
| Alcohol | 0.04 | 0.03 | 0.22 | 0.01 | 0.03 | 0.80 | 0.06 | 0.03 | 0.07 |
| Dietary Quality Score | **Est** | **SE** | ***p*** | **Est** | **SE** | ***p*** | **Est** | **SE** | ***p*** |
| Alternate Healthy Eating Index | **0.09** | **0.03** | **0.002** | 0.01 | 0.03 | 0.75 | -0.04 | 0.03 | 0.23 |
| Mediterranean Diet Score | 0.04 | 0.03 | 0.13 | 0.03 | 0.03 | 0.40 | 0.0001 | 0.03 | 0.10 |

Dirichlet regression models adjusted for age, sex, energy intake, education, smoking, and physical activity; n=1442; significant values in **bold** p<0.05

*=Significant after adjustment with Bonferroni correction (0.05/39=0.00128)

**Additional Table 2.** Associations between dietary factors (per SD) and microbial subgroups in the gut

|  | Subgroup 4 | | | | Subgroup 5 | | | | Subgroup 6 | | | |
| --- | --- | --- | --- | --- | --- | --- | --- | --- | --- | --- | --- | --- |
| Food Item | **Est** | **SE** | ***p*** | **Est** | | **SE** | ***p*** | **Est** | | **SE** | ***p*** |  |
| Potatoes | 0.003 | 0.03 | 0.99 | 0.03 | | 0.03 | 0.39 | -0.02 | | 0.03 | 0.59 |  |
| Vegetables | -0.02 | 0.03 | 0.59 | **0.10** | | **0.03** | **0.00113*** | -0.02 | | 0.03 | 0.60 |  |
| Legumes | 0.0004 | 0.03 | 0.99 | **0.08** | | **0.02** | **0.0009*** | 0.01 | | 0.03 | 0.74 |  |
| Fruit | -0.01 | 0.03 | 0.66 | **0.10** | | **0.03** | **0.0003*** | **-0.07** | | **0.03** | **0.01** |  |
| Nuts and seeds | -0.02 | 0.03 | 0.54 | **0.08** | | **0.02** | **0.0008*** | 0.04 | | 0.03 | 0.09 |  |
| Dairy products | 0.02 | 0.03 | 0.61 | **0.07** | | **0.02** | **0.006** | -0.04 | | 0.03 | 0.17 |  |
| Yogurt | -0.01 | 0.03 | 0.62 | 0.04 | | 0.02 | 0.07 | 0.004 | | 0.03 | 0.87 |  |
| Cheese | 0.01 | 0.03 | 0.86 | **0.06** | | **0.03** | **0.03** | -0.03 | | 0.03 | 0.35 |  |
| (Refined) Grains | -0.0002 | 0.04 | 1.00 | **-0.10** | | **0.04** | **0.007** | -0.01 | | 0.04 | 0.81 |  |
| Whole grains | -0.03 | 0.03 | 0.30 | **0.09** | | **0.03** | **0.0003*** | -0.05 | | 0.03 | 0.05 |  |
| Fresh red meat | 0.01 | 0.04 | 0.86 | 0.03 | | 0.04 | 0.37 | -0.004 | | 0.04 | 0.92 |  |
| Processed (red) meat | 0.02 | 0.03 | 0.57 | -0.05 | | 0.03 | 0.13 | -0.004 | | 0.03 | 0.92 |  |
| Fish and shellfish | 0.02 | 0.03 | 0.39 | 0.05 | | 0.03 | 0.06 | -0.005 | | 0.03 | 0.87 |  |
| Eggs | 0.01 | 0.03 | 0.85 | -0.007 | | 0.03 | 0.77 | 0.01 | | 0.03 | 0.65 |  |
| Animal fats | -0.004 | 0.03 | 0.89 | **-0.11** | | **0.03** | **0.0001*** | 0.003 | | 0.03 | 0.92 |  |
| Plant oils | -0.01 | 0.03 | 0.78 | **0.09** | | **0.03** | **0.00097*** | 0.03 | | 0.03 | 0.22 |  |
| Sugar and sweets | -0.02 | 0.03 | 0.61 | 0.03 | | 0.03 | 0.28 | 0.05 | | 0.03 | 0.08 |  |
| Cakes | -0.01 | 0.03 | 0.76 | -0.05 | | 0.03 | 0.12 | -0.03 | | 0.03 | 0.29 |  |
| Coffee | 0.02 | 0.03 | 0.48 | **0.07** | | **0.02** | **0.007** | 0.01 | | 0.03 | 0.81 |  |
| SSB | 0.01 | 0.03 | 0.61 | **-0.09** | | **0.03** | **0.00127*** | 0.004 | | 0.03 | 0.89 |  |
| Wine | 0.02 | 0.03 | 0.41 | 0.01 | | 0.02 | 0.57 | -0.003 | | 0.03 | 0.90 |  |
| Beer | 0.02 | 0.03 | 0.50 | **-0.10** | | **0.03** | **0.0009*** | 0.04 | | 0.03 | 0.23 |  |
| Nutrient | **Est** | **SE** | ***p*** | **Est** | | **SE** | ***p*** | **Est** | | **SE** | ***p*** |  |
| Total fat | -0.02 | 0.06 | 0.73 | -0.002 | | 0.06 | 0.98 | 0.10 | | 0.06 | 0.11 |  |
| Total carbohydrates | -0.05 | 0.07 | 0.43 | 0.05 | | 0.06 | 0.39 | -0.10 | | 0.07 | 0.12 |  |
| Total protein | 0.05 | 0.05 | 0.35 | **0.19** | | **0.05** | **0.0002*** | -0.04 | | 0.05 | 0.41 |  |
| Total fiber | -0.05 | 0.04 | 0.23 | **0.15** | | **0.03** | **7.38E-06*** | **-0.10** | | **0.04** | **0.02** |  |
| Soluble fiber | -0.04 | 0.04 | 0.27 | **0.11** | | **0.03** | **0.002** | **-0.11** | | **0.04** | **0.009** |  |
| Insoluble fiber | -0.05 | 0.04 | 0.22 | **0.16** | | **0.03** | **6.49E-07*** | **-0.08** | | **0.04** | **0.03** |  |
| Alcohol | 0.03 | 0.03 | 0.29 | **-0.09** | | **0.03** | **0.004** | 0.03 | | 0.03 | 0.34 |  |
| Dietary Quality Score | **Est** | **SE** | ***p*** | **Est** | | **SE** | ***p*** | **Est** | | **SE** | ***p*** |  |
| Alternate Healthy Eating Index | -0.02 | 0.03 | 0.52 | **0.14** | | **0.03** | **3.57E-07*** | -0.02 | | 0.03 | 0.41 |  |
| Mediterranean Diet Score | 0.01 | 0.03 | 0.79 | **0.12** | | **0.03** | **4.33E-05*** | -0.05 | | 0.03 | 0.09 |  |

Dirichlet regression models adjusted for age, sex, energy intake, education, smoking, and physical activity; n=1442; significant values in **bold** p<0.05

*=Significant after adjustment with Bonferroni correction (0.05/39=0.00128)

**Additional Table 2.** Associations between dietary factors (per SD) and microbial subgroups in the gut

|  | Subgroup 7 | | | | Subgroup 8 | | | | Subgroup 9 | | | |
| --- | --- | --- | --- | --- | --- | --- | --- | --- | --- | --- | --- | --- |
| Food Item | **Est** | **SE** | ***p*** | **Est** | | **SE** | ***p*** | **Est** | | **SE** | ***p*** |  |
| Potatoes | 0.02 | 0.03 | 0.53 | -0.01 | | 0.03 | 0.87 | 0.05 | | 0.03 | 0.13 |  |
| Vegetables | 0.02 | 0.03 | 0.48 | -0.001 | | 0.03 | 0.97 | -0.005 | | 0.03 | 0.88 |  |
| Legumes | 0.03 | 0.03 | 0.25 | -0.01 | | 0.03 | 0.69 | -0.01 | | 0.03 | 0.80 |  |
| Fruit | -0.03 | 0.03 | 0.27 | 0.003 | | 0.03 | 0.91 | 0.004 | | 0.03 | 0.89 |  |
| Nuts and seeds | **0.05** | **0.03** | **0.05** | 0.002 | | 0.03 | 0.95 | -0.01 | | 0.03 | 0.65 |  |
| Dairy products | -0.03 | 0.03 | 0.21 | -0.001 | | 0.03 | 0.97 | 0.01 | | 0.03 | 0.61 |  |
| Yogurt | -0.02 | 0.03 | 0.54 | 0.01 | | 0.03 | 0.83 | -0.02 | | 0.03 | 0.44 |  |
| Cheese | 0.01 | 0.03 | 0.64 | 0.001 | | 0.03 | 0.97 | -0.02 | | 0.03 | 0.44 |  |
| (Refined) Grains | 0.004 | 0.04 | 0.91 | 0.01 | | 0.04 | 0.86 | -0.003 | | 0.04 | 0.95 |  |
| Whole grains | -0.001 | 0.03 | 0.97 | 0.003 | | 0.03 | 0.93 | -0.002 | | 0.03 | 0.94 |  |
| Fresh red meat | 0.05 | 0.04 | 0.21 | 0.01 | | 0.04 | 0.88 | 0.03 | | 0.04 | 0.43 |  |
| Processed (red) meat | 0.01 | 0.03 | 0.86 | -0.01 | | 0.03 | 0.70 | 0.03 | | 0.03 | 0.37 |  |
| Fish and shellfish | 0.02 | 0.03 | 0.48 | -0.001 | | 0.03 | 0.96 | 0.01 | | 0.03 | 0.86 |  |
| Eggs | -0.02 | 0.02 | 0.48 | -0.01 | | 0.03 | 0.83 | 0.02 | | 0.03 | 0.43 |  |
| Animal fats | 0.02 | 0.03 | 0.40 | -0.02 | | 0.03 | 0.62 | 0.01 | | 0.03 | 0.73 |  |
| Plant oils | 0.03 | 0.03 | 0.23 | 0.005 | | 0.03 | 0.87 | -0.01 | | 0.03 | 0.76 |  |
| Sugar and sweets | 0.02 | 0.03 | 0.55 | -0.005 | | 0.03 | 0.87 | -0.05 | | 0.03 | 0.08 |  |
| Cakes | **-0.09** | **0.03** | **0.003** | -0.005 | | 0.03 | 0.88 | -0.01 | | 0.03 | 0.79 |  |
| Coffee | **0.08** | **0.03** | **0.00098*** | 0.02 | | 0.03 | 0.38 | -0.02 | | 0.03 | 0.36 |  |
| SSB | **-0.08** | **0.03** | **0.003** | -0.01 | | 0.03 | 0.86 | -0.01 | | 0.03 | 0.77 |  |
| Wine | **0.07** | **0.02** | **0.0026** | 0.01 | | 0.03 | 0.82 | -0.002 | | 0.03 | 0.95 |  |
| Beer | **0.06** | **0.03** | **0.049** | -0.004 | | 0.03 | 0.90 | 0.03 | | 0.03 | 0.44 |  |
| Nutrient | **Est** | **SE** | ***p*** | **Est** | | **SE** | ***p*** | **Est** | | **SE** | ***p*** |  |
| Total fat | 0.05 | 0.06 | 0.42 | -0.03 | | 0.06 | 0.64 | 0.01 | | 0.06 | 0.92 |  |
| Total carbohydrates | **-0.18** | **0.06** | **0.002** | 0.02 | | 0.07 | 0.72 | -0.04 | | 0.07 | 0.52 |  |
| Total protein | 0.06 | 0.05 | 0.20 | 0.01 | | 0.05 | 0.89 | 0.02 | | 0.05 | 0.77 |  |
| Total fiber | -0.03 | 0.04 | 0.47 | 0.001 | | 0.04 | 0.97 | 0.02 | | 0.04 | 0.69 |  |
| Soluble fiber | -0.05 | 0.04 | 0.19 | -0.002 | | 0.04 | 0.96 | 0.03 | | 0.04 | 0.40 |  |
| Insoluble fiber | -0.02 | 0.04 | 0.56 | 0.003 | | 0.04 | 0.94 | 0.01 | | 0.04 | 0.87 |  |
| Alcohol | **0.09** | **0.03** | **0.003** | -0.0003 | | 0.03 | 0.99 | 0.02 | | 0.03 | 0.48 |  |
| Dietary Quality Score | **Est** | **SE** | ***p*** | **Est** | | **SE** | ***p*** | **Est** | | **SE** | ***p*** |  |
| Alternate Healthy Eating Index | 0.03 | 0.03 | 0.34 | -0.002 | | 0.03 | 0.94 | -0.004 | | 0.03 | 0.90 |  |
| Mediterranean Diet Score | 0.03 | 0.03 | 0.23 | -0.003 | | 0.03 | 0.93 | 0.02 | | 0.03 | 0.60 |  |

Dirichlet regression models adjusted for age, sex, energy intake, education, smoking, and physical activity; n=1442; significant values in **bold** p<0.05

*=Significant after adjustment with Bonferroni correction (0.05/39=0.00128)

**Additional Table 2.** Associations between dietary factors (per SD) and microbial subgroups in the gut

|  | Subgroup 10 | | | Subgroup 11 | | | Subgroup 12 | | |
| --- | --- | --- | --- | --- | --- | --- | --- | --- | --- |
| Food Item | **Est** | **SE** | ***p*** | **Est** | **SE** | ***p*** | **Est** | **SE** | ***p*** |
| Potatoes | -0.01 | 0.03 | 0.68 | -0.02 | 0.03 | 0.55 | 0.01 | 0.03 | 0.73 |
| Vegetables | -0.02 | 0.03 | 0.45 | -0.001 | 0.03 | 0.98 | -0.01 | 0.03 | 0.68 |
| Legumes | 0.01 | 0.03 | 0.82 | -0.01 | 0.03 | 0.84 | -0.01 | 0.03 | 0.75 |
| Fruit | -0.02 | 0.03 | 0.44 | 0.03 | 0.03 | 0.27 | **-0.07** | **0.03** | **0.01** |
| Nuts and seeds | -0.01 | 0.03 | 0.72 | -0.02 | 0.03 | 0.52 | -0.05 | 0.03 | 0.10 |
| Dairy products | -0.02 | 0.03 | 0.38 | 0.02 | 0.03 | 0.51 | -0.02 | 0.03 | 0.43 |
| Yogurt | -0.02 | 0.03 | 0.46 | -0.004 | 0.03 | 0.89 | -0.003 | 0.03 | 0.91 |
| Cheese | -0.001 | 0.03 | 0.97 | 0.004 | 0.03 | 0.89 | 0.05 | 0.03 | 0.07 |
| (Refined) Grains | 0.01 | 0.04 | 0.85 | -0.01 | 0.04 | 0.85 | **0.07** | **0.04** | **0.04** |
| Whole grains | -0.03 | 0.03 | 0.30 | 0.01 | 0.03 | 0.64 | -0.05 | 0.03 | 0.06 |
| Fresh red meat | 0.004 | 0.04 | 0.92 | 0.02 | 0.04 | 0.55 | -0.02 | 0.04 | 0.59 |
| Processed (red) meat | 0.02 | 0.03 | 0.64 | 0.02 | 0.03 | 0.56 | -0.004 | 0.03 | 0.90 |
| Fish and shellfish | -0.01 | 0.03 | 0.80 | 0.01 | 0.03 | 0.83 | -0.03 | 0.03 | 0.22 |
| Eggs | 0.01 | 0.03 | 0.84 | -0.01 | 0.03 | 0.64 | -0.01 | 0.03 | 0.72 |
| Animal fats | 0.02 | 0.03 | 0.54 | -0.001 | 0.03 | 0.99 | -0.03 | 0.03 | 0.24 |
| Plant oils | -0.01 | 0.03 | 0.86 | -0.01 | 0.03 | 0.61 | 0.02 | 0.03 | 0.38 |
| Sugar and sweets | -0.01 | 0.03 | 0.77 | -0.05 | 0.03 | 0.11 | 0.03 | 0.03 | 0.21 |
| Cakes | 0.02 | 0.03 | 0.54 | -0.002 | 0.03 | 0.94 | -0.06 | 0.03 | 0.07 |
| Coffee | 0.02 | 0.03 | 0.46 | 0.004 | 0.03 | 0.88 | **0.06** | **0.02** | **0.019** |
| SSB | 0.02 | 0.03 | 0.48 | 0.01 | 0.03 | 0.78 | 0.03 | 0.03 | 0.24 |
| Wine | 0.03 | 0.03 | 0.24 | 0.002 | 0.03 | 0.94 | -0.004 | 0.03 | 0.87 |
| Beer | 0.01 | 0.03 | 0.69 | 0.01 | 0.03 | 0.81 | **0.06** | **0.03** | **0.04** |
| Nutrient | **Est** | **SE** | ***p*** | **Est** | **SE** | ***p*** | **Est** | **SE** | ***p*** |
| Total fat | 0.03 | 0.06 | 0.58 | -0.05 | 0.06 | 0.47 | -0.08 | 0.06 | 0.17 |
| Total carbohydrates | -0.06 | 0.06 | 0.34 | 0.02 | 0.06 | 0.73 | 0.01 | 0.06 | 0.92 |
| Total protein | -0.02 | 0.05 | 0.70 | 0.01 | 0.05 | 0.79 | -0.04 | 0.05 | 0.47 |
| Total fiber | -0.05 | 0.04 | 0.17 | 0.02 | 0.04 | 0.59 | **-0.10** | **0.04** | **0.006** |
| Soluble fiber | -0.05 | 0.04 | 0.27 | 0.02 | 0.04 | 0.56 | **-0.07** | **0.04** | **0.047** |
| Insoluble fiber | -0.05 | 0.04 | 0.17 | 0.02 | 0.04 | 0.57 | **-0.11** | **0.04** | **0.002** |
| Alcohol | 0.03 | 0.03 | 0.31 | 0.01 | 0.03 | 0.84 | 0.06 | 0.03 | 0.06 |
| Dietary Quality Score | **Est** | **SE** | ***p*** | **Est** | **SE** | ***p*** | **Est** | **SE** | ***p*** |
| Alternate Healthy Eating Index | -0.02 | 0.03 | 0.42 | -0.02 | 0.03 | 0.52 | **-0.09** | **0.03** | **0.0009*** |
| Mediterranean Diet Score | 0.01 | 0.03 | 0.85 | 0.01 | 0.03 | 0.72 | -0.05 | 0.03 | 0.10 |

Dirichlet regression models adjusted for age, sex, energy intake, education, smoking, and physical activity; n=1442; significant values in **bold** p<0.05

*=Significant after adjustment with Bonferroni correction (0.05/39=0.00128)

**Additional Table 2.** Associations between dietary factors (per SD) and microbial subgroups in the gut

|  | Subgroup 13 | | | Subgroup 14 | | | Subgroup 15 | | |
| --- | --- | --- | --- | --- | --- | --- | --- | --- | --- |
| Food Item | **Est** | **SE** | ***p*** | **Est** | **SE** | ***p*** | **Est** | **SE** | ***p*** |
| Potatoes | -0.04 | 0.03 | 0.25 | -0.01 | 0.03 | 0.83 | -0.01 | 0.03 | 0.78 |
| Vegetables | -0.04 | 0.03 | 0.20 | 0.06 | 0.03 | 0.05 | -0.02 | 0.03 | 0.56 |
| Legumes | 0.04 | 0.03 | 0.16 | 0.02 | 0.03 | 0.46 | 0.01 | 0.03 | 0.73 |
| Fruit | -0.03 | 0.03 | 0.26 | **0.12** | **0.03** | **0.00004*** | -0.03 | 0.03 | 0.38 |
| Nuts and seeds | **0.06** | **0.02** | **0.01** | **0.06** | **0.03** | **0.01** | -0.04 | 0.03 | 0.20 |
| Dairy products | 0.02 | 0.02 | 0.31 | **0.06** | **0.03** | **0.02** | 0.01 | 0.03 | 0.80 |
| Yogurt | -0.02 | 0.02 | 0.53 | **0.08** | **0.03** | **0.005** | 0.01 | 0.03 | 0.63 |
| Cheese | 0.02 | 0.03 | 0.44 | **0.10** | **0.03** | **0.0003*** | 0.02 | 0.03 | 0.52 |
| (Refined) Grains | -0.03 | 0.03 | 0.38 | 0.01 | 0.04 | 0.75 | 0.03 | 0.04 | 0.39 |
| Whole grains | -0.02 | 0.02 | 0.39 | **0.13** | **0.03** | **0.000001*** | -0.01 | 0.03 | 0.66 |
| Fresh red meat | 0.01 | 0.04 | 0.72 | **-0.10** | **0.04** | **0.01** | 0.03 | 0.04 | 0.53 |
| Processed (red) meat | 0.002 | 0.03 | 0.94 | **-0.14** | **0.03** | **0.00004*** | -0.01 | 0.03 | 0.84 |
| Fish and shellfish | 0.02 | 0.02 | 0.50 | 0.04 | 0.03 | 0.10 | 0.02 | 0.03 | 0.52 |
| Eggs | 0.02 | 0.02 | 0.41 | **-0.08** | **0.03** | **0.002** | 0.03 | 0.03 | 0.31 |
| Animal fats | -0.02 | 0.03 | 0.54 | -0.04 | 0.03 | 0.17 | -0.001 | 0.03 | 0.98 |
| Plant oils | 0.05 | 0.03 | 0.06 | 0.01 | 0.03 | 0.82 | 0.01 | 0.03 | 0.69 |
| Sugar and sweets | **0.07** | **0.03** | **0.004** | -0.01 | 0.03 | 0.76 | 0.01 | 0.03 | 0.83 |
| Cakes | -0.02 | 0.03 | 0.40 | -0.001 | 0.03 | 0.97 | 0.001 | 0.03 | 0.97 |
| Coffee | 0.03 | 0.02 | 0.14 | 0.04 | 0.03 | 0.15 | 0.02 | 0.03 | 0.36 |
| SSB | **-0.06** | **0.02** | **0.025** | **-0.07** | **0.03** | **0.018** | 0.01 | 0.03 | 0.80 |
| Wine | 0.01 | 0.02 | 0.53 | 0.04 | 0.02 | 0.075 | 0.01 | 0.03 | 0.68 |
| Beer | -0.01 | 0.03 | 0.75 | **-0.07** | **0.03** | **0.03** | 0.003 | 0.03 | 0.94 |
| Nutrient | **Est** | **SE** | ***p*** | **Est** | **SE** | ***p*** | **Est** | **SE** | ***p*** |
| Total fat | **0.12** | **0.06** | **0.03** | **-0.18** | **0.06** | **0.003** | -0.01 | 0.06 | 0.82 |
| Total carbohydrates | **-0.14** | **0.06** | **0.02** | **0.16** | **0.06** | **0.01** | -0.03 | 0.06 | 0.60 |
| Total protein | **0.10** | **0.05** | **0.04** | 0.09 | 0.05 | 0.09 | 0.06 | 0.05 | 0.26 |
| Total fiber | **-0.08** | **0.03** | **0.02** | **0.25** | **0.04** | **1.29E-11*** | -0.05 | 0.04 | 0.19 |
| Soluble fiber | **-0.09** | **0.03** | **0.0095** | **0.23** | **0.04** | **2.21E-09*** | -0.04 | 0.04 | 0.36 |
| Insoluble fiber | **-0.08** | **0.03** | **0.02** | **0.25** | **0.04** | **1.03E-11*** | -0.05 | 0.04 | 0.16 |
| Alcohol | -0.001 | 0.03 | 0.96 | -0.04 | 0.03 | 0.25 | 0.01 | 0.03 | 0.66 |
| Dietary Quality Score | **Est** | **SE** | ***p*** | **Est** | **SE** | ***p*** | **Est** | **SE** | ***p*** |
| Alternate Healthy Eating Index | 0.01 | 0.03 | 0.59 | **0.20** | **0.03** | **1.94E-11*** | -0.02 | 0.03 | 0.43 |
| Mediterranean Diet Score | -0.04 | 0.03 | 0.13 | **0.09** | **0.03** | **0.002** | 0.02 | 0.03 | 0.60 |

Dirichlet regression models adjusted for age, sex, energy intake, education, smoking, and physical activity; n=1442; significant values in **bold** p<0.05

*=Significant after adjustment with Bonferroni correction (0.05/39=0.00128)

**Additional Table 2.** Associations between dietary factors (per SD) and microbial subgroups in the gut

|  | Subgroup 16 | | | Subgroup 17 | | | Subgroup 18 | | |
| --- | --- | --- | --- | --- | --- | --- | --- | --- | --- |
| Food Item | **Est** | **SE** | ***p*** | **Est** | **SE** | ***p*** | **Est** | **SE** | ***p*** |
| Potatoes | -0.04 | 0.03 | 0.27 | 0.002 | 0.03 | 0.94 | -0.002 | 0.03 | 0.95 |
| Vegetables | -0.01 | 0.03 | 0.77 | 0.04 | 0.03 | 0.22 | **-0.08** | **0.03** | **0.007** |
| Legumes | 0.02 | 0.03 | 0.45 | 0.02 | 0.03 | 0.39 | -0.02 | 0.03 | 0.54 |
| Fruit | -0.02 | 0.03 | 0.46 | 0.05 | 0.03 | 0.13 | -0.06 | 0.03 | 0.06 |
| Nuts and seeds | 0.02 | 0.03 | 0.51 | 0.03 | 0.03 | 0.23 | -0.01 | 0.03 | 0.60 |
| Dairy products | 0.02 | 0.03 | 0.43 | 0.01 | 0.03 | 0.60 | 0.01 | 0.03 | 0.76 |
| Yogurt | -0.02 | 0.03 | 0.49 | 0.02 | 0.03 | 0.46 | -0.01 | 0.03 | 0.83 |
| Cheese | **0.05** | **0.03** | **0.049** | 0.01 | 0.03 | 0.75 | -0.04 | 0.03 | 0.20 |
| (Refined) Grains | 0.03 | 0.04 | 0.41 | -0.02 | 0.04 | 0.62 | 0.03 | 0.04 | 0.44 |
| Whole grains | **0.09** | **0.03** | **0.0008*** | 0.04 | 0.03 | 0.14 | -0.03 | 0.03 | 0.26 |
| Fresh red meat | 0.01 | 0.04 | 0.76 | -0.01 | 0.04 | 0.80 | 0.002 | 0.04 | 0.96 |
| Processed (red) meat | -0.05 | 0.03 | 0.15 | -0.03 | 0.03 | 0.44 | -0.05 | 0.03 | 0.11 |
| Fish and shellfish | -0.01 | 0.03 | 0.65 | 0.02 | 0.03 | 0.52 | 0.01 | 0.03 | 0.66 |
| Eggs | -0.04 | 0.03 | 0.11 | 0.02 | 0.03 | 0.48 | 0.02 | 0.03 | 0.51 |
| Animal fats | -0.01 | 0.03 | 0.69 | -0.001 | 0.03 | 0.98 | 0.03 | 0.03 | 0.39 |
| Plant oils | **0.07** | **0.03** | **0.014** | 0.003 | 0.03 | 0.93 | -0.002 | 0.03 | 0.95 |
| Sugar and sweets | **0.08** | **0.03** | **0.0019** | 0.03 | 0.03 | 0.33 | **0.09** | **0.03** | **0.0008*** |
| Cakes | **0.08** | **0.03** | **0.007** | -0.03 | 0.03 | 0.41 | **0.08** | **0.03** | **0.01** |
| Coffee | **0.11** | **0.03** | **0.00001*** | 0.02 | 0.03 | 0.49 | 0.03 | 0.03 | 0.24 |
| SSB | **-0.08** | **0.03** | **0.0026** | -0.003 | 0.03 | 0.91 | 0.01 | 0.03 | 0.61 |
| Wine | 0.03 | 0.02 | 0.16 | 0.04 | 0.03 | 0.12 | 0.02 | 0.03 | 0.38 |
| Beer | **-0.08** | **0.03** | **0.007** | -0.05 | 0.03 | 0.14 | **-0.07** | **0.03** | **0.02** |
| Nutrient | **Est** | **SE** | ***p*** | **Est** | **SE** | ***p*** | **Est** | **SE** | ***p*** |
| Total fat | 0.05 | 0.06 | 0.40 | -0.05 | 0.06 | 0.45 | 0.02 | 0.06 | 0.80 |
| Total carbohydrates | 0.05 | 0.06 | 0.47 | 0.04 | 0.06 | 0.54 | 0.09 | 0.07 | 0.20 |
| Total protein | 0.01 | 0.05 | 0.77 | 0.03 | 0.05 | 0.56 | -0.08 | 0.05 | 0.12 |
| Total fiber | 0.03 | 0.03 | 0.37 | 0.07 | 0.04 | 0.06 | -0.07 | 0.04 | 0.06 |
| Soluble fiber | 0.03 | 0.03 | 0.40 | 0.05 | 0.04 | 0.17 | -0.05 | 0.04 | 0.17 |
| Insoluble fiber | 0.03 | 0.03 | 0.37 | **0.07** | **0.04** | **0.046** | **-0.08** | **0.04** | **0.04** |
| Alcohol | -0.06 | 0.03 | 0.07 | -0.01 | 0.03 | 0.70 | -0.04 | 0.03 | 0.23 |
| Dietary Quality Score | **Est** | **SE** | ***p*** | **Est** | **SE** | ***p*** | **Est** | **SE** | ***p*** |
| Alternate Healthy Eating Index | 0.03 | 0.03 | 0.26 | 0.05 | 0.03 | 0.09 | -0.02 | 0.03 | 0.44 |
| Mediterranean Diet Score | 0.004 | 0.03 | 0.88 | 0.05 | 0.03 | 0.10 | -0.04 | 0.03 | 0.24 |

Dirichlet regression models adjusted for age, sex, energy intake, education, smoking, and physical activity; n=1442; significant values in **bold** p<0.05

*=Significant after adjustment with Bonferroni correction (0.05/39=0.00128)

**Additional Table 2.** Associations between dietary factors (per SD) and microbial subgroups in the gut

|  | Subgroup 19 | | | | Subgroup 20 | | |
| --- | --- | --- | --- | --- | --- | --- | --- |
| Food Item | **Est** | **SE** | ***p*** | **Est** | | **SE** | ***p*** |
| Potatoes | 0.02 | 0.03 | 0.50 | 0.01 | | 0.03 | 0.88 |
| Vegetables | 0.03 | 0.03 | 0.29 | -0.001 | | 0.03 | 0.99 |
| Legumes | 0.01 | 0.03 | 0.69 | -0.03 | | 0.03 | 0.27 |
| Fruit | -0.02 | 0.03 | 0.60 | 0.01 | | 0.03 | 0.84 |
| Nuts and seeds | -0.02 | 0.03 | 0.45 | -0.01 | | 0.03 | 0.71 |
| Dairy products | 0.02 | 0.03 | 0.45 | **0.07** | | **0.03** | **0.01** |
| Yogurt | 0.01 | 0.03 | 0.81 | -0.003 | | 0.03 | 0.90 |
| Cheese | 0.01 | 0.03 | 0.73 | 0.05 | | 0.03 | 0.06 |
| (Refined) Grains | -0.01 | 0.04 | 0.89 | 0.05 | | 0.04 | 0.22 |
| Whole grains | -0.01 | 0.03 | 0.80 | **0.06** | | **0.03** | **0.02** |
| Fresh red meat | -0.01 | 0.04 | 0.84 | 0.01 | | 0.04 | 0.72 |
| Processed (red) meat | 0.03 | 0.03 | 0.32 | **-0.10** | | **0.03** | **0.004** |
| Fish and shellfish | 0.01 | 0.03 | 0.65 | 0.01 | | 0.03 | 0.79 |
| Eggs | 0.01 | 0.03 | 0.76 | -0.05 | | 0.03 | 0.10 |
| Animal fats | 0.01 | 0.03 | 0.72 | 0.00002 | | 0.03 | 1.00 |
| Plant oils | -0.01 | 0.03 | 0.81 | 0.01 | | 0.03 | 0.74 |
| Sugar and sweets | 0.004 | 0.03 | 0.89 | **0.09** | | **0.03** | **0.002** |
| Cakes | 0.01 | 0.03 | 0.65 | **0.09** | | **0.03** | **0.003** |
| Coffee | -0.004 | 0.03 | 0.88 | 0.02 | | 0.03 | 0.52 |
| SSB | 0.02 | 0.03 | 0.50 | -0.04 | | 0.03 | 0.20 |
| Wine | -0.004 | 0.03 | 0.90 | 0.04 | | 0.02 | 0.13 |
| Beer | -0.04 | 0.03 | 0.22 | **-0.10** | | **0.03** | **0.0019** |
| Nutrient | **Est** | **SE** | ***p*** | **Est** | | **SE** | ***p*** |
| Total fat | 0.07 | 0.06 | 0.28 | -0.04 | | 0.06 | 0.50 |
| Total carbohydrates | -0.02 | 0.07 | 0.73 | **0.13** | | **0.06** | **0.04** |
| Total protein | 0.05 | 0.05 | 0.40 | -0.01 | | 0.05 | 0.87 |
| Total fiber | -0.02 | 0.04 | 0.60 | **0.09** | | **0.04** | **0.01** |
| Soluble fiber | -0.01 | 0.04 | 0.74 | **0.14** | | **0.04** | **0.0006*** |
| Insoluble fiber | -0.02 | 0.04 | 0.59 | **0.08** | | **0.04** | **0.03** |
| Alcohol | -0.04 | 0.03 | 0.25 | **-0.06** | | **0.03** | **0.046** |
| Dietary Quality Score | **Est** | **SE** | ***p*** | **Est** | | **SE** | ***p*** |
| Alternate Healthy Eating Index | -0.03 | 0.03 | 0.35 | **0.07** | | **0.03** | **0.02** |
| Mediterranean Diet Score | -0.01 | 0.03 | 0.82 | 0.003 | | 0.03 | 0.93 |

Dirichlet regression models adjusted for age, sex, energy intake, education, smoking, and physical activity; n=1442; significant values in **bold** p<0.05

*=Significant after adjustment with Bonferroni correction (0.05/39=0.00128)
