# Supplementary material for: Associations between habitual diet, metabolic disease, and the gut microbiota using latent Dirichlet allocation
Source: Microbiome. 2021 Mar 16;9:61. doi: 10.1186/s40168-020-00969-9 (PMC7967986; doi:10.1186/s40168-020-00969-9)
Supplement: Supplementary file 4 — Additional file 3. [file 40168_2020_969_MOESM4_ESM.docx]

**Additional Table 3.** Associations between metabolic diseases or risk factors and microbial sub-communities in the gut

|  | | Subgroup 1 | | | | Subgroup 2 | | | Subgroup 3 | | | Subgroup 4 | | | Subgroup 5 | | |
| --- | --- | --- | --- | --- | --- | --- | --- | --- | --- | --- | --- | --- | --- | --- | --- | --- | --- |
| Disease or  Risk Factor | **Sample Size** | **Est** | **SE** | ***p*** | **Est** | | **SE** | ***p*** | **Est** | **SE** | ***p*** | **Est** | **SE** | ***p*** | **Est** | **SE** | ***p*** |
| BMI^a^ | 1990 | **-0.09** | **0.02** | **2.80E^-05^*** | -0.005 | | 0.02 | 0.83 | 0.04 | 0.02 | 0.11 | 0.006 | 0.02 | 0.79 | -0.001 | 0.02 | 0.97 |
| WC^a^ | 1990 | **-0.11** | **0.02** | **1.65E^-05^*** | -0.004 | | 0.03 | 0.88 | 0.04 | 0.03 | 0.08 | 0.02 | 0.03 | 0.53 | -0.04 | 0.02 | 0.11 |
| HDL-c^a,b^ | 1972 | **0.10** | **0.02** | **2.15^E-05^*** | 0.01 | | 0.03 | 0.63 | -0.004 | 0.02 | 0.88 | -0.005 | 0.03 | 0.85 | 0.04 | 0.02 | 0.09 |
| LDL-c^a,b^ | 1972 | -0.03 | -0.03 | 0.22 | -0.01 | | 0.02 | 0.65 | -0.02 | 0.02 | 0.49 | 0.002 | 0.02 | 0.93 | **0.07** | **0.02** | **0.0014** |
| Total cholesterol^a,b^ | 1972 | -0.004 | 0.02 | 0.87 | -0.007 | | 0.02 | 0.75 | -0.01 | 0.02 | 0.62 | -0.003 | 0.02 | 0.91 | **0.06** | **0.02** | **0.01** |
| Triglycerides^a,b^ | 1972 | **-0.08** | **0.02** | **0.0004*** | -0.02 | | 0.02 | 0.34 | 0.01 | 0.02 | 0.65 | 0.003 | 0.02 | 0.90 | **-0.11** | **0.02** | **1.76^E-06^*** |
| Diabetes^c^ | 1976 |  |  |  |  | |  |  |  |  |  |  |  |  |  |  |  |
| *Prediabetes* | *679* | **-0.13** | **0.05** | **0.01** | 0.04 | | 0.05 | 0.45 | 0.08 | 0.05 | 0.14 | 0.02 | 0.05 | 0.66 | -0.06 | 0.05 | 0.20 |
| *UDM* | *83* | **-0.31** | **0.11** | **0.01** | 0.07 | | 0.12 | 0.53 | 0.04 | 0.12 | 0.73 | -3.75^E-04^ | 0.12 | 1.00 | -0.09 | 0.11 | 0.41 |
| *Prevalent T2DM* | *199* | **-0.15** | **0.08** | **0.05** | -0.01 | | 0.08 | 0.95 | **0.17** | **0.08** | **0.04** | 0.02 | 0.08 | 0.85 | **-0.39** | **0.08** | **5.22^E-07^*** |
| *Unknown/Other* | *66* | -0.05 | 0.12 | 0.67 | 0.07 | | 0.13 | 0.60 | -0.08 | 0.13 | 0.50 | 0.09 | 0.13 | 0.48 | 0.06 | 0.12 | 0.63 |
| Hypertension^d^ | 1989 |  |  |  |  | |  |  |  |  |  |  |  |  |  |  |  |
| *Controlled* | *521* | 0.01 | 0.05 | 0.81 | -0.05 | | 0.06 | 0.43 | 0.07 | 0.06 | 0.21 | -0.01 | 0.06 | 0.90 | -0.09 | 0.05 | 0.10 |
| *Uncontrolled* | *136* | -0.07 | 0.09 | 0.46 | 0.05 | | 0.09 | 0.62 | 0.03 | 0.09 | 0.77 | -0.02 | 0.09 | 0.85 | -0.14 | 0.09 | 0.11 |
| *Untreated* | *80* | 0.13 | 0.11 | 0.23 | -0.03 | | 0.11 | 0.77 | 0.18 | 0.11 | 0.11 | 0.02 | 0.11 | 0.85 | 0.06 | 0.11 | 0.59 |
| *Undiagnosed* | *50* | 0.15 | 0.14 | 0.27 | 0.02 | | 0.14 | 0.86 | 0.09 | 0.14 | 0.51 | -0.06 | 0.14 | 0.68 | -0.24 | 0.14 | 0.08 |

HDL-c, high-denisty lipoprotein cholesterol; LDL-c, low-denisty lipoprotein cholesterol; BMI, body mass index; WC, waist circumference; UDM, undetected diabetes mellitus; T2DM, type 2 diabetes mellitus.
Dirichlet regression models adjusted for age, sex, education, smoking, and physical activity; significant values in **bold** p<0.05

^a^Effect size given per standard deviation

^b^Additionally adjusted for use of lipid-lowering medication

^c^Normal glucose tolerance as reference (n=949)

^d^Normal blood pressure as reference (n=1202)

*=Significant after adjustment with Bonferroni correction (0.05/39=0.00128)

**Additional Table 3.** Associations between metabolic diseases or risk factors and microbial sub-communities in the gut

|  | | Subgroup 6 | | | Subgroup 7 | | | Subgroup 8 | | | Subgroup 9 | | | Subgroup 10 | | |
| --- | --- | --- | --- | --- | --- | --- | --- | --- | --- | --- | --- | --- | --- | --- | --- | --- |
| Disease or  Risk Factor | **Sample Size** | **Est** | **SE** | ***p*** | **Est** | **SE** | ***p*** | **Est** | **SE** | ***p*** | **Est** | **SE** | ***p*** | **Est** | **SE** | ***p*** |
| BMI^a^ | 1990 | 0.03 | 0.02 | 0.23 | 0.04 | 0.02 | 0.06 | -0.007 | 0.02 | 0.78 | 0.02 | 0.02 | 0.41 | -0.02 | 0.02 | 0.49 |
| WC^a^ | 1990 | 0.04 | 0.02 | 0.08 | 0.02 | 0.02 | 0.33 | -0.01 | 0.03 | 0.67 | 0.02 | 0.03 | 0.45 | -0.005 | 0.03 | 0.84 |
| HDL-c^a,b^ | 1972 | -0.02 | 0.024 | 0.39 | -0.015 | 0.02 | 0.51 | -0.002 | 0.03 | 0.95 | 0.04 | 0.03 | 0.16 | 0.04 | 0.02 | 0.14 |
| LDL-c^a,b^ | 1972 | 0.02 | 0.02 | 0.37 | **0.07** | **0.02** | **0.003** | 0.02 | 0.02 | 0.47 | 0.001 | 0.02 | 0.97 | -0.02 | 0.02 | 0.34 |
| Total cholesterol^a,b^ | 1972 | 0.02 | 0.02 | 0.33 | **0.07** | **0.02** | **0.002** | 0.02 | 0.02 | 0.52 | 0.004 | 0.02 | 0.88 | -0.02 | 0.02 | 0.35 |
| Triglycerides^a,b^ | 1972 | **0.10** | **0.02** | **1.26^E-05^*** | **0.05** | **0.02** | **0.03** | -0.005 | 0.02 | 0.83 | -0.03 | 0.02 | 0.17 | -0.04 | 0.02 | 0.07 |
| Diabetes^c^ | 1976 |  |  |  |  |  |  |  |  |  |  |  |  |  |  |  |
| *Prediabetes* | *679* | 0.01 | 0.05 | 0.86 | **0.11** | **0.05** | **0.03** | -0.01 | 0.05 | 0.90 | 0.03 | 0.05 | 0.63 | -0.05 | 0.05 | 0.30 |
| *UDM* | *83* | 0.05 | 0.11 | 0.64 | -0.15 | 0.08 | 0.06 | -0.03 | 0.12 | 0.81 | -0.01 | 0.12 | 0.94 | -0.02 | 0.12 | 0.84 |
| *Prevalent T2DM* | *199* | 0.12 | 0.08 | 0.13 | -0.15 | 0.08 | 0.06 | -0.06 | 0.08 | 0.50 | -0.06 | 0.08 | 0.46 | -0.06 | 0.08 | 0.49 |
| *Unknown/Other* | *66* | 0.05 | 0.12 | 0.68 | -0.02 | 0.12 | 0.86 | 0.02 | 0.13 | 0.89 | -0.01 | 0.13 | 0.93 | -0.15 | 0.13 | 0.23 |
| Hypertension^d^ | 1989 |  |  |  |  |  |  |  |  |  |  |  |  |  |  |  |
| *Controlled* | *521* | 0.06 | 0.06 | 0.30 | -0.03 | 0.05 | 0.64 | 0.003 | 0.06 | 0.97 | -0.004 | 0.06 | 0.95 | 0.01 | 0.06 | 0.90 |
| *Uncontrolled* | *136* | 0.16 | 0.09 | 0.08 | 0.04 | 0.09 | 0.62 | 0.01 | 0.09 | 0.88 | -0.03 | 0.09 | 0.73 | -0.06 | 0.09 | 0.52 |
| *Untreated* | *80* | -0.06 | 0.11 | 0.62 | 0.01 | 0.11 | 0.91 | -0.02 | 0.12 | 0.84 | 0.03 | 0.11 | 0.78 | 0.08 | 0.11 | 0.49 |
| *Undiagnosed* | *50* | 0.26 | 0.14 | 0.06 | **0.28** | **0.13** | **0.03** | 0.02 | 0.14 | 0.91 | 0.03 | 0.14 | 0.85 | -0.03 | 0.14 | 0.81 |

HDL-c, high-denisty lipoprotein cholesterol; LDL-c, low-denisty lipoprotein cholesterol; BMI, body mass index; WC, waist circumference; UDM, undetected diabetes mellitus; T2DM, type 2 diabetes mellitus.
Dirichlet regression models adjusted for age, sex, education, smoking, and physical activity; significant values in **bold** p<0.05

^a^Effect size given per standard deviation

^b^Additionally adjusted for use of lipid-lowering medication

^c^Normal glucose tolerance as reference (n=949)

^d^Normal blood pressure as reference (n=1202)

*=Significant after adjustment with Bonferroni correction (0.05/39=0.00128)

**Additional Table 3.** Associations between metabolic diseases or risk factors and microbial sub-communities in the gut

|  | | Subgroup 11 | | | Subgroup 12 | | | Subgroup 13 | | | Subgroup 14 | | | Subgroup 15 | | |
| --- | --- | --- | --- | --- | --- | --- | --- | --- | --- | --- | --- | --- | --- | --- | --- | --- |
| Disease or  Risk Factor | **Sample Size** | **Est** | **SE** | ***p*** | **Est** | **SE** | ***p*** | **Est** | **SE** | ***p*** | **Est** | **SE** | ***p*** | **Est** | **SE** | ***p*** |
| BMI^a^ | 1990 | 0.01 | 0.02 | 0.66 | 0.01 | 0.02 | 0.62 | **0.07** | **0.02** | **5.12E^-04^*** | **-0.12** | **0.02** | **1.22E^-06^*** | 0.01 | 0.02 | 0.54 |
| WC^a^ | 1990 | 0.0001 | 0.03 | 1.00 | 0.01 | 0.02 | 0.65 | **0.07** | **0.02** | **0.002** | **-0.15** | **0.03** | **1.17E^-08^*** | 0.02 | 0.03 | 0.40 |
| HDL-c^a,b^ | 1972 | 0.02 | 0.03 | 0.49 | 0.04 | 0.02 | 0.09 | 0.01 | 0.02 | 0.55 | **0.09** | **0.02** | **0.0002*** | 0.006 | 0.03 | 0.82 |
| LDL-c^a,b^ | 1972 | 0.02 | 0.02 | 0.45 | 0.02 | 0.02 | 0.32 | 0.04 | 0.02 | 0.11 | 0.002 | 0.02 | 0.92 | 0.02 | 0.02 | 0.40 |
| Total cholesterol^a,b^ | 1972 | 0.02 | 0.02 | 0.43 | **0.07** | **0.02** | **0.003** | **0.05** | **0.02** | **0.03** | 0.005 | 0.02 | 0.82 | 0.03 | 0.02 | 0.30 |
| Triglycerides^a,b^ | 1972 | -0.03 | 0.02 | 0.18 | 0.03 | 0.02 | 0.13 | 0.04 | 0.02 | 0.07 | **-0.13** | **0.03** | **2.64^E-07^*** | -0.01 | 0.02 | 0.71 |
| Diabetes^c^ | 1976 |  |  |  |  |  |  |  |  |  |  |  |  |  |  |  |
| *Prediabetes* | *679* | 0.04 | 0.05 | 0.47 | **0.10** | **0.05** | **0.04** | **0.11** | **0.05** | **0.03** | -0.08 | 0.05 | 0.13 | -0.02 | 0.05 | 0.66 |
| *UDM* | *83* | -0.05 | 0.12 | 0.65 | -0.01 | 0.11 | 0.90 | -0.06 | 0.11 | 0.59 | **-0.37** | **0.12** | **0.0016** | -0.004 | 0.12 | 0.97 |
| *Prevalent T2DM* | *199* | -0.05 | 0.08 | 0.54 | -0.07 | 0.08 | 0.37 | 0.13 | 0.08 | 0.09 | **-0.29** | **0.08** | **5.31^E-04^*** | 0.001 | 0.08 | 0.99 |
| *Unknown/Other* | *66* | 0.03 | 0.13 | 0.78 | 0.09 | 0.12 | 0.46 | 0.18 | 0.11 | 0.11 | -0.21 | 0.13 | 0.09 | 2.16^E-04^ | 0.13 | 0.99 |
| Hypertension^d^ | 1989 |  |  |  |  |  |  |  |  |  |  |  |  |  |  |  |
| *Controlled* | *521* | -0.01 | 0.06 | 0.86 | 0.02 | 0.05 | 0.78 | **0.13** | **0.05** | **0.01** | **-0.17** | **0.06** | **0.003** | 0.03 | 0.06 | 0.58 |
| *Uncontrolled* | *136* | -0.02 | 0.09 | 0.79 | 0.01 | 0.09 | 0.92 | 0.09 | 0.08 | 0.27 | **-0.20** | **0.09** | **0.03** | 0.04 | 0.09 | 0.70 |
| *Untreated* | *80* | -0.05 | 0.11 | 0.69 | 0.16 | 0.11 | 0.14 | 0.16 | 0.10 | 0.11 | 0.01 | 0.11 | 0.95 | -0.001 | 0.11 | 0.99 |
| *Undiagnosed* | *50* | -0.05 | 0.14 | 0.75 | 0.04 | 0.14 | 0.78 | **0.32** | **0.13** | **0.01** | -0.24 | 0.14 | 0.10 | -0.08 | 0.14 | 0.59 |

HDL-c, high-denisty lipoprotein cholesterol; LDL-c, low-denisty lipoprotein cholesterol; BMI, body mass index; WC, waist circumference; UDM, undetected diabetes mellitus; T2DM, type 2 diabetes mellitus.
Dirichlet regression models adjusted for age, sex, education, smoking, and physical activity; significant values in **bold** p<0.05

^a^Effect size given per standard deviation

^b^Additionally adjusted for use of lipid-lowering medication

^c^Normal glucose tolerance as reference (n=949)

^d^Normal blood pressure as reference (n=1202)

*=Significant after adjustment with Bonferroni correction (0.05/39=0.00128)

**Additional Table 3.** Associations between metabolic diseases or risk factors and microbial sub-communities in the gut

|  | | Subgroup 16 | | | Subgroup 17 | | | Subgroup 18 | | | Subgroup 19 | | | Subgroup 20 | | |
| --- | --- | --- | --- | --- | --- | --- | --- | --- | --- | --- | --- | --- | --- | --- | --- | --- |
| Disease or  Risk Factor | **Sample Size** | **Est** | **SE** | ***p*** | **Est** | **SE** | ***p*** | **Est** | **SE** | ***p*** | **Est** | **SE** | ***p*** | **Est** | **SE** | ***p*** |
| BMI^a^ | 1990 | **-0.05** | **0.02** | **0.02** | -0.04 | 0.02 | 0.10 | **-0.08** | **0.02** | **2.82E^-04^*** | 0.02 | 0.02 | 0.46 | **-0.13** | **0.02** | **5.11E^-08^*** |
| WC^a^ | 1990 | **-0.08** | **0.02** | **0.0006*** | -0.05 | -0.05 | 0.07 | **-0.09** | **-0.16** | **3.18E^-04^*** | 0.03 | -0.16 | 0.29 | **-0.16** | **0.03** | **7.12E^-10^*** |
| HDL-c^a,b^ | 1972 | **0.07** | **0.02** | **0.002** | 0.009 | 0.02 | 0.71 | **0.05** | **0.02** | **0.04** | -0.023 | 0.03 | 0.24 | **0.11** | **0.02** | **8.32^E-06^*** |
| LDL-c^a,b^ | 1972 | 0.02 | 0.02 | 0.38 | 0.009 | 0.02 | 0.70 | **-0.05** | **0.02** | **0.04** | -0.03 | 0.02 | 0.16 | -0.04 | 0.02 | 0.07 |
| Total cholesterol^a,b^ | 1972 | 0.01 | 0.02 | 0.62 | 0.009 | 0.02 | 0.72 | -0.04 | 0.02 | 0.10 | -0.04 | 0.02 | 0.14 | -0.04 | 0.02 | 0.13 |
| Triglycerides^a,b^ | 1972 | **-0.13** | **0.02** | **2.63^E-08^*** | -0.03 | 0.02 | 0.24 | **-0.06** | **0.02** | **0.009** | 0.03 | 0.02 | 0.24 | **-0.14** | **0.03** | **3.39^E-08^*** |
| Diabetes^c^ | 1976 |  |  |  |  |  |  |  |  |  |  |  |  |  |  |  |
| *Prediabetes* | *679* | -0.07 | 0.05 | 0.13 | -0.004 | 0.05 | 0.93 | **-0.09** | **0.05** | **0.09** | 0.01 | 0.05 | 0.83 | -0.08 | 0.05 | 0.14 |
| *UDM* | *83* | -0.16 | 0.11 | 0.15 | -0.02 | 0.12 | 0.89 | -0.04 | 0.11 | 0.71 | 0.07 | 0.12 | 0.53 | **-0.23** | **0.12** | **0.04** |
| *Prevalent T2DM* | *199* | **-0.33** | **0.08** | **3.53^E-05^*** | -0.08 | 0.08 | 0.34 | 0.01 | 0.08 | 0.89 | **0.42** | **0.08** | **2.00^E-07^*** | **-0.17** | **0.08** | **0.04** |
| *Unknown/Other* | *66* | 0.04 | 0.12 | 0.75 | 0.01 | 0.13 | 0.92 | -0.18 | 0.12 | 0.14 | 0.07 | 0.13 | 0.60 | -0.22 | 0.12 | 0.07 |
| Hypertension^d^ | 1989 |  |  |  |  |  |  |  |  |  |  |  |  |  |  |  |
| *Controlled* | *521* | **-0.11** | **0.05** | **0.047** | -0.04 | 0.06 | 0.48 | -9.56E^-05^ | 0.06 | 1.00 | -0.03 | 0.06 | 0.54 | **-0.16** | **0.06** | **0.005** |
| *Uncontrolled* | *136* | -0.14 | 0.09 | 0.11 | -0.08 | 0.09 | 0.36 | -0.07 | 0.09 | 0.43 | 0.06 | 0.09 | 0.51 | **-0.30** | **0.09** | **0.0008*** |
| *Untreated* | *80* | -0.07 | 0.11 | 0.53 | 0.07 | 0.11 | 0.52 | -0.08 | 0.11 | 0.50 | -0.07 | 0.11 | 0.52 | -0.13 | 0.11 | 0.23 |
| *Undiagnosed* | *50* | -0.03 | 0.14 | 0.81 | -0.06 | 0.14 | 0.69 | 0.09 | 0.14 | 0.54 | 0.05 | 0.14 | 0.70 | 0.08 | 0.14 | 0.57 |

HDL-c, high-denisty lipoprotein cholesterol; LDL-c, low-denisty lipoprotein cholesterol; BMI, body mass index; WC, waist circumference; UDM, undetected diabetes mellitus; T2DM, type 2 diabetes mellitus.
Dirichlet regression models adjusted for age, sex, education, smoking, and physical activity; significant values in **bold** p<0.05

^a^Effect size given per standard deviation

^b^Additionally adjusted for use of lipid-lowering medication

^c^Normal glucose tolerance as reference (n=949)

^d^Normal blood pressure as reference (n=1202)

*=Significant after adjustment with Bonferroni correction (0.05/39=0.00128)
