# Supplementary material for: Associations between habitual diet, metabolic disease, and the gut microbiota using latent Dirichlet allocation
Source: Microbiome. 2021 Mar 16;9:61. doi: 10.1186/s40168-020-00969-9 (PMC7967986; doi:10.1186/s40168-020-00969-9)
Supplement: Supplementary file 6 — Additional file 5. [file 40168_2020_969_MOESM6_ESM.pdf]

Code for the analysis *Associations between habitual diet, metabolic disease, and the gut microbiota using Latent Dirichlet Allocation*

Breuninger et al.

This is the complete R code used for the analysis "Associations between habitual diet, metabolic disease, and the gut microbiota using Latent Dirichlet Allocation" by Breuninger et al.

In the code, the subgroups are referred to as "topics," as this is common terminology used with Latent Dirichlet Allocation (LDA).

First, we load the data sets containing diet, disease, and covariate information for the participants. We remove any participants taking antibiotics (n=41) and for whom microbiome data is not available (n=1).

```
df_cov <- read.csv("K01918g_Jones_FF4_tra20180626_kopie.csv", dec = ".", as.is = T,
    header = T)
# 2034 observations

df_cov2 <- read.csv("K01918g_Jones_FF4_E1_tra20190529_kopie.csv", dec = ".", as.is = T,
    header = T)
# 2034 observations

# merge
df_covall <- merge(df_cov, df_cov2, by = "zz_nr_ff4_seq_darm", all = T)

# applying exclusion criteria
table(df_covall$u3tmabio_j01, useNA = "always")
df_covall <- subset(df_covall, u3tmabio_j01 != 1, )  #40 removed
table(df_covall$u3tmabio_j01, useNA = "always")
table(df_covall$u3tmabio_son, useNA = "always")
df_covall <- subset(df_covall, u3tmabio_son != 1, )  #1 more removed
table(df_covall$u3tmabio_son, useNA = "always")  #1993 left


# load OTU table
otu <- read.csv2("zz_otu_kopie.csv", sep = ";", dec = ",", as.is = T, header = T)
# 2033 observations
otu$X <- NULL


# remove observations not in df_covall
otu_ex <- otu[otu$zz_nr_ff4_seq_darm %in% df_covall$zz_nr_ff4_seq_darm, ]
# remove participants taking abx, 1992 left
NA.row <- otu_ex[rowSums(is.na(otu_ex)) > 0, ]  #No NAs

Nutribiome <- df_covall[df_covall$zz_nr_ff4_seq_darm %in% otu_ex$zz_nr_ff4_seq_darm,
```

```
    ]  #removing extra person in df_covall, now 1992
```

Then we calculate the descriptive statistics used in Table 1 (participant characteristics).

```
# ex: age - repeated for each continuous variable
(tapply(Nutribiome$u3talteru, Nutribiome$u3csex, mean, na.rm = TRUE))
(tapply(Nutribiome$u3talteru, Nutribiome$u3csex, sd, na.rm = TRUE))

mean(Nutribiome$u3talteru)
sd(Nutribiome$u3talteru)


# ex: education - repeated for each categorical variable
round((prop.table(table(Nutribiome$ed2, Nutribiome$u3csex, useNA = "always"), margin = 2)) *
    100, 1)
table(Nutribiome$ed2, Nutribiome$u3csex, useNA = "always")

round((prop.table(table(Nutribiome$ed2, useNA = "always"))) * 100, 1)
table(Nutribiome$ed2, useNA = "always")
```

And the descriptive statistics for Table 2 (habitual diet)

```
Nutribiome_Nutr <- Nutribiome[complete.cases(Nutribiome$u3v_e01), ]

# ex: vegetables - repeated for each nutrition variable
(tapply(Nutribiome_Nutr$u3v_e02, Nutribiome_Nutr$u3csex, mean, na.rm = TRUE))
(tapply(Nutribiome_Nutr$u3v_e02, Nutribiome_Nutr$u3csex, sd, na.rm = TRUE))

mean(Nutribiome_Nutr$u3v_e02)
sd(Nutribiome_Nutr$u3v_e02)
```

We then filter the OTU table with 0.1% abundance and 1% persistance, leaving 1713 OTUs remaining

```
library("OTUtable")

abundance <- data.frame(otu_ex[, -1], row.names = otu_ex[, 1])
t_abundance <- t(abundance)
t_filtered_abundance <- data.frame(filter_taxa(t_abundance, abundance = 0.1, persistence = 1),
    check.names = F)
filtered_abundance <- data.frame(t(t_filtered_abundance))
filtered_abundance1 <- filtered_abundance
```

Our OTU table contains relative abundances. The LDA function from the package MetaTopics requires a matrix of integers as an input. Here we multiply the relative abundances by 1000 to preserve decimal places and transform the OTU table to a matrix containing integers.

```
filtered_abundance_matrix1 <- filtered_abundance1
filtered_abundance_matrix1[, 1:1713] <- filtered_abundance_matrix1[, 1:1713] * 1000
filtered_abundance_matrix1[, 1:1713] <- sapply(filtered_abundance_matrix1[1:1713],
    as.integer)
filtered_abundance_matrix1 <- as.matrix(filtered_abundance_matrix1)
```

Now that the matrix is properly formatted, we can select the number of subgroups (k) for LDA. We tested models with 15-110 subgroups (by steps of 5) and also 130-180 subgroups (by steps of 10). There was no clear optimum subgroup number, as perplexity and loglikelihood simply improved with increasing k. A small jump in performance was seen around 15-25 subgroups, and finally k=20 was chosen after comparing model results.

```
library(slam)
library(topicmodels)
install.packages("devtools", dependencies = TRUE, repos = "http://cran.rstudio.com/")
require(devtools)
install_github("bm2-lab/MetaTopics")
library(MetaTopics)

# set parameters
dtm = as.simple_triplet_matrix(filtered_abundance_matrix1)
seed_num = 2019
fold_num = 3
kv_num = c(10, 15, 20, 25)  #topic numbers to compare
sp = smp(cross = fold_num, n = nrow(dtm), seed = seed_num)
control = list(seed = seed_num, burnin = 100, thin = 10, iter = 100)

ctmK = selectK(dtm = dtm, kv = kv_num, SEED = seed_num, cross = fold_num, sp = sp,
    method = "Gibbs", control = control)

# compare results
plot_perplexity(ctmK, kv_num)
```

Now that we have chosen the number of subgroups, we fit 5 LDA models with different seed numbers.

```
# set parameters
dtm = as.simple_triplet_matrix(filtered_abundance_matrix1)
control = list(seed = seed_num, burnin = 1000, thin = 100, iter = 1000)

# 1st model
seed_num = 2019
Gibbs_model_example = LDA(dtm, k = 20, method = "Gibbs", control = list(seed = seed_num,
    burnin = 1000, thin = 100, iter = 1000))

# 2nd model
seed_num = 1234
Gibbs_model_example2 = LDA(dtm, k = 20, method = "Gibbs", control = list(seed = seed_num,
    burnin = 1000, thin = 100, iter = 1000))

# 3rd model
seed_num = 1600
Gibbs_model_example3 = LDA(dtm, k = 20, method = "Gibbs", control = list(seed = seed_num,
    burnin = 1000, thin = 100, iter = 1000))


# 4th model
seed_num = 3232
Gibbs_model_example4 = LDA(dtm, k = 20, method = "Gibbs", control = list(seed = seed_num,
    burnin = 1000, thin = 100, iter = 1000))
```

```
# 5th model
seed_num = 5432
Gibbs_model_example5 = LDA(dtm, k = 20, method = "Gibbs", control = list(seed = seed_num,
    burnin = 1000, thin = 100, iter = 1000))
```

Here we extract the top 10 OTUs for each subgroup for each of the 5 models and export them as an excel file to evaluate.

```
# extracting top 10 OTUs in each topic

terms1 <- terms(Gibbs_model_example, k = 10)
terms2 <- terms(Gibbs_model_example2, k = 10)
terms3 <- terms(Gibbs_model_example3, k = 10)
terms4 <- terms(Gibbs_model_example4, k = 5)
terms5 <- terms(Gibbs_model_example5, k = 10)

library(xlsx)

terms1 <- t(terms1)
write.xlsx(terms1, file = "LDA_20_1713.xlsx", sheetName = "Model 1", append = FALSE)

terms2 <- t(terms2)
write.xlsx(terms2, file = "LDA_20_1713.xlsx", sheetName = "Model 2", append = TRUE)

terms3 <- t(terms3)
write.xlsx(terms3, file = "LDA_20_1713.xlsx", sheetName = "Model 3", append = TRUE)

terms4 <- t(terms4)
write.xlsx(terms4, file = "LDA_20_1713.xlsx", sheetName = "Model 4", append = TRUE)

terms5 <- t(terms5)
write.xlsx(terms5, file = "LDA_20_1713.xlsx", sheetName = "Model 5", append = TRUE)
```

After comparison, Model 4 was selected. We then extract the probabilities from the LDA output for each individual for each of the 20 subgroups and add it to the data set containing all other variables.

```
LDA_20_1_df <- as.data.frame(Gibbs_model_example4@gamma)

names(LDA_20_1_df) <- c("Topic_1", "Topic_2", "Topic_3", "Topic_4", "Topic_5", "Topic_6",
    "Topic_7", "Topic_8", "Topic_9", "Topic_10", "Topic_11", "Topic_12", "Topic_13",
    "Topic_14", "Topic_15", "Topic_16", "Topic_17", "Topic_18", "Topic_19", "Topic_20")

LDA_20_1_df$zz_nr_ff4_seq_darm <- Gibbs_model_example4@documents
LDA_20_1_df$zz_nr_ff4_seq_darm <- as.integer(LDA_20_1_df$zz_nr_ff4_seq_darm)

Nutribiome_Topics <- merge(Nutribiome, LDA_20_1_df, by = "zz_nr_ff4_seq_darm", all = T)
```

We then extract all OTUs with a probability of 1% for any subgroup and export these top 99% OTUs for each subgroup to an excel file. This was used to produce Figure 1 and Additional File 1.

#fix hidden code below

```r
my_beta<- exp(Gibbs_model_example4@beta)
colnames(my_beta) <- Gibbs_model_example4@terms
max(my_beta)
min(my_beta)
rowSums(my_beta) #all add up to 1
mybeta_df <- as.data.frame(my_beta)
# df with probabilities for all 1713 OTUs for all 20 Topics


library(dplyr)

#extract OTUs present >= 1% in any topic
mybeta_dfselect <- mybeta_df %>%  select_if(~max(., na.rm = TRUE) >= 0.01)
# df with probabilities for all 251 OTUs >=1% for all 20 Topics

rowSums(mybeta_dfselect)
max(mybeta_dfselect)


#repeat code below for Topics 1-20
T20 <- mybeta_dfselect[20,] %>%
  select_if(~any(. >= 0.01))
T20 <- sort(unlist(T20), decreasing=T)
T20 <- as.data.frame(t(T20))


#save results in excel file

write.xlsx(T1, file = "OTUsperTopic.xlsx", #for writing first sheet
           sheetName="Topic 1", append=FALSE)

write.xlsx(T20, file = "OTUsperTopic.xlsx", #for adding all additional sheets
            sheetName="Topic 2", append=TRUE)
```

Now we want to describe the topics. We create a violin plot showing the distribution of each subgroup in the study population (Figure 2).

```r
library(dplyr)
```

```
##
## Attaching package: 'dplyr'

## The following objects are masked from 'package:stats':
##
##     filter, lag

## The following objects are masked from 'package:base':
##
##     intersect, setdiff, setequal, union
```

```r
library(tidyr)
library(forcats)
library(hrbrthemes)
```

```
## NOTE: Either Arial Narrow or Roboto Condensed fonts are required to use these themes.

##       Please use hrbrthemes::import_roboto_condensed() to install Roboto Condensed and

##       if Arial Narrow is not on your system, please see https://bit.ly/arialnarrow
```

```r
library(viridis)
```

```
## Loading required package: viridisLite
```

```r
library(ggplot2)


Topics_df <- Nutribiome_Topics[, c(1, 331:350)]
datalong <- reshape(Topics_df, varying = list(names(Topics_df)[2:21]), timevar = "Topic",
    v.names = "Probability", idvar = "zz_nr_ff4_seq_darm", direction = "long", sep = "_")

datalong$Topic <- as.factor(datalong$Topic)

(p <- datalong %>% mutate(name = fct_relevel(Topic, "1", "2", "3", "4", "5", "6",
    "7", "8", "9", "10", "11", "12", "13", "14", "15", "16", "17", "18", "19", "20")) %>%
    ggplot(aes(x = Topic, y = Probability, fill = Topic, color = Topic)) + geom_violin(scale = "width",
    trim = T) + scale_fill_viridis(discrete = TRUE, direction = 1) + geom_boxplot(width = 0.07,
    fill = "white") + theme_bw() + stat_summary(fun.y = median, geom = "point", fill = "white",
    shape = 21, size = 1.25) + scale_color_viridis(discrete = TRUE, , direction = 1) +
    scale_x_discrete(limits = rev(levels(Topics_df$Topic))) + theme(legend.position = "none") +
    xlab("Subgroup") + ylab("Assigned Probability"))
```

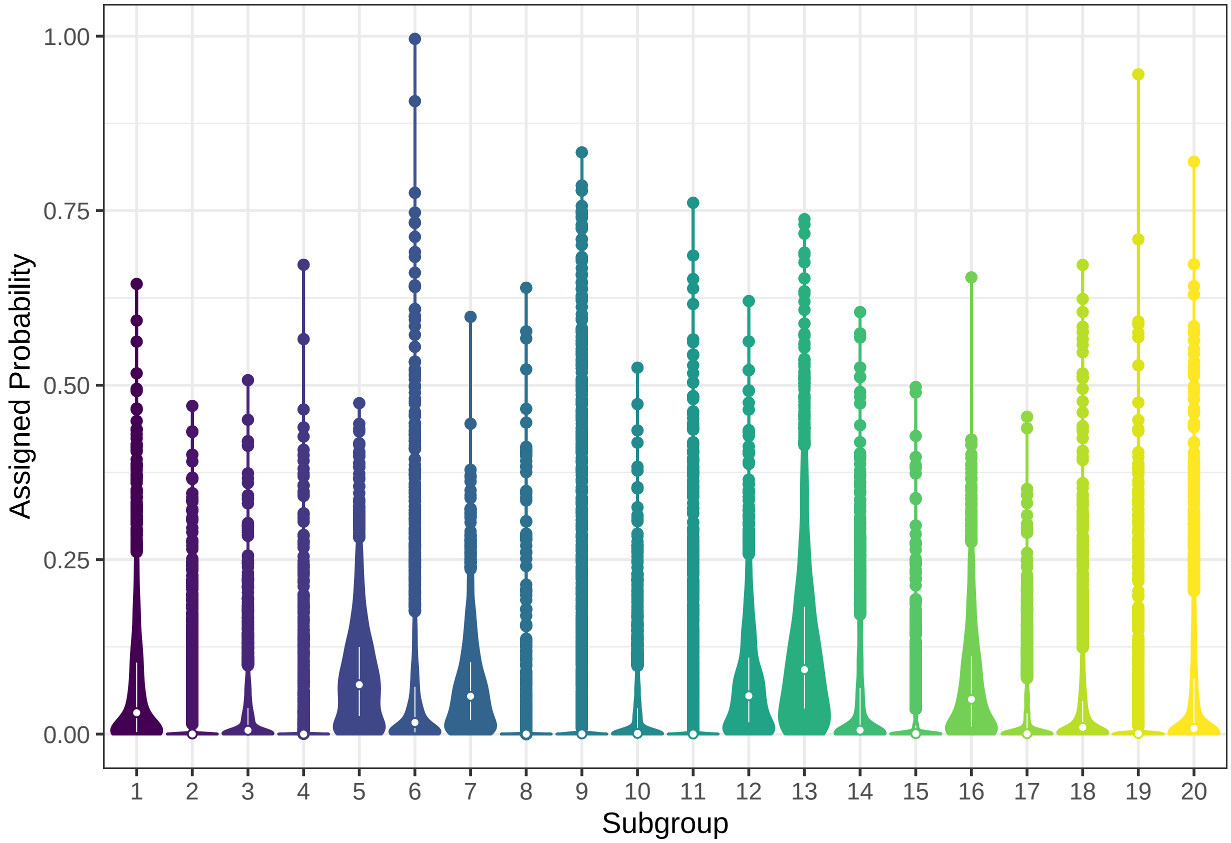

We want to calculate some more descriptive statistics regarding the subgroups. Here we determine what the maximum probability each person has for their most prominent subgroup, and then determine how many subgroups a person has on average with more than 1% probability, more than 10%, 25%, and so on.

```
library("matrixStats")
```

```
##
## Attaching package: 'matrixStats'

## The following object is masked from 'package:dplyr':
##
##     count
```

```
matrix.Gibbs <- as.matrix(Gibbs_model_example4@gamma)


probability_max1 <- rowMaxs(matrix.Gibbs, value = FALSE)
# vector containing max probabilites for each person

names(probability_max1) <- Gibbs_model_example4@documents

min(probability_max1)  #13.1%
```

```
## [1] 0.1312071
```

```r
max(probability_max1)  #99.6%
```

```
## [1] 0.9961574
```

```r
mean(probability_max1)  #33.24%
```

```
## [1] 0.3323944
```

```r
sd(probability_max1)  #1.27%
```

```
## [1] 0.1270371
```

```r
median(probability_max1)  #30.4%
```

```
## [1] 0.3034776
```

```r
# getting mean and sd per cut-off#

df.Gibbs <- as.data.frame(matrix.Gibbs)
df.Gibbs$Max <- rowMaxs(matrix.Gibbs, value = FALSE)


subgroupsover0.5 <- rowSums(df.Gibbs > 0.05)
subgroupsover1 <- rowSums(df.Gibbs > 0.01)
subgroupsover5 <- rowSums(df.Gibbs > 0.5)
subgroupsover10 <- rowSums(df.Gibbs > 0.1)
subgroupsover25 <- rowSums(df.Gibbs > 0.25)


# repeat below code for mean and sd
mean(subgroupsover0.5)  #11.02 +/- 2.4 subgroups >0.5%
```

```
## [1] 6.856928
```

```r
mean(subgroupsover1)  #average of 10 +/- 2.2 Subgroups >1% per person
```

```
## [1] 10.26305
```

```r
mean(subgroupsover5)  #6.9 +/- 1.6 subgroups >5%
```

```
## [1] 0.2259036
```

```r
mean(subgroupsover10)  #4.5 +/- 1.1 subgroups >10%
```

```
## [1] 4.486948
```

```
mean(subgroupsover25)  #1.5 +/- 1.1 subgroups >25%
```

```
## [1] 1.542671
```

To better visualize this, we produce a histogram displaying the maximum subgroup probability. This gives us a feeling for how common it is for one subgroup to mostly describe a participant's microbiota, or for the subgroups to more evenly contribute to a participant's microbiota. (Here we see that both cases are rather rare.)

```
library(ggplot2)
library(viridis)


(plot <- ggplot(df.Gibbs, aes(x = Max)) + geom_histogram(aes(fill = ..count..), binwidth = 0.02) +
    theme_bw() + scale_fill_viridis(begin = 0, end = 0.3, direction = -1) + scale_x_continuous(name = "[illegible]
    breaks = seq(0, 1, 0.1)) + scale_y_continuous(name = "Count", breaks = seq(0,
    200, 25), limits = c(0, 200)))
```

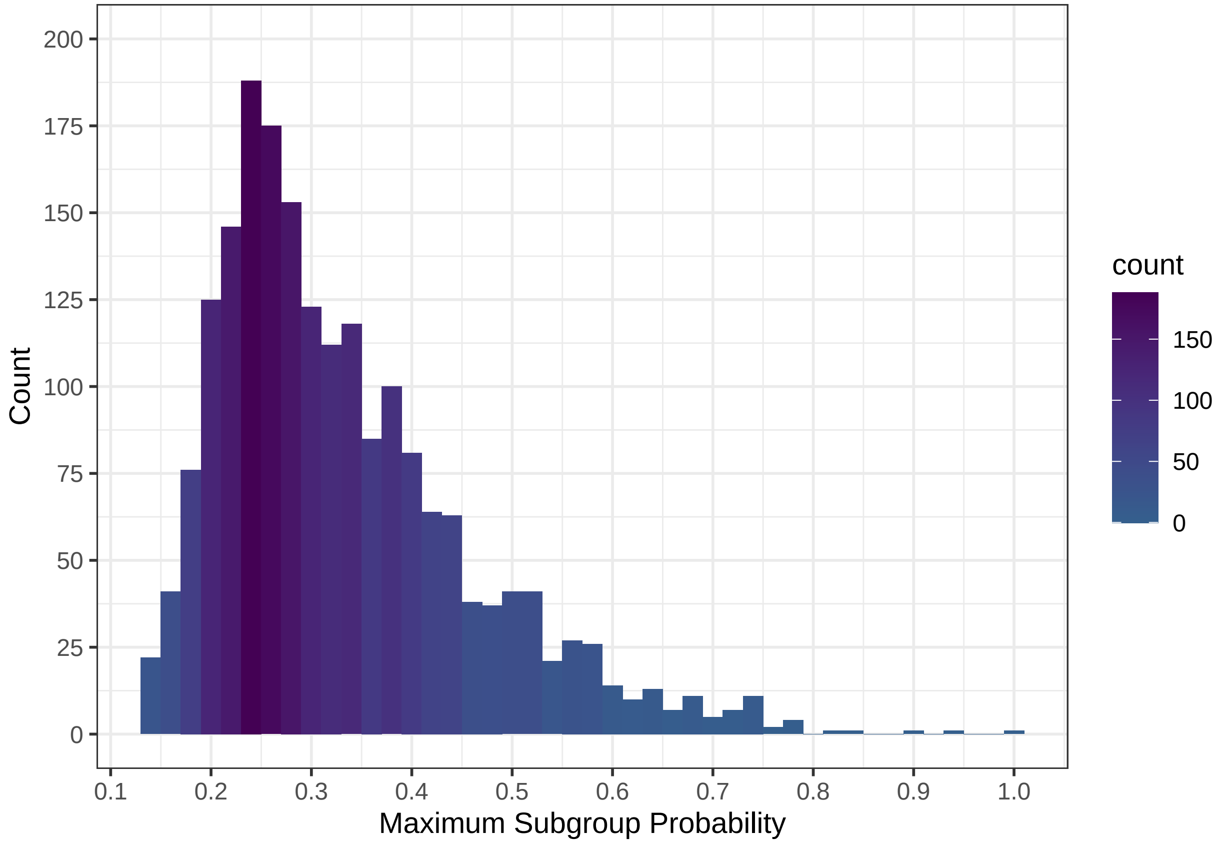

We want to find out if there is an association between long-term diet and the subgroups. Here we use Dirichlet regressions since they can model all 20 subgroups together as one outcome. First we divide all diet variables by their sd.

```
#ex: vegetables - repeat for each nutr variable
(sd.vegetables <- sd(Nutribiome_Nutr$u3v_e02, na.rm = T))
Nutribiome_Nutr$vegetables.sd <- Nutribiome_Nutr$u3v_e02/sd.vegetables
```

Then we fit the Dirichlet regressions, checking the effects of different levels of covariate adjustment and if the fit improves with a quadratic effect. Model 3 has the best fit. One model is fitted for each food, nutrient, and diet quality score.

```
library("DirichletReg")

Topics_matrix <- DR_data(Nutribiome_Nutr[, c("Topic_1", "Topic_2", "Topic_3", "Topic_4",
    "Topic_5", "Topic_6", "Topic_7", "Topic_8", "Topic_9", "Topic_10", "Topic_11",
    "Topic_12", "Topic_13", "Topic_14", "Topic_15", "Topic_16", "Topic_17", "Topic_18",
    "Topic_19", "Topic_20")], trafo = F)

# any missings in covariates?
table(Nutribiome_Nutr$u3csex, useNA = "always")
sum(is.na(Nutribiome_Nutr$u3talteru))
sum(is.na(Nutribiome_Nutr$u3v_gj))
sum(is.na(Nutribiome_Nutr$ed2))
sum(is.na(Nutribiome_Nutr$u3tcigsmk))
sum(is.na(Nutribiome_Nutr$u3tphys))
# all complete


# fit the model
model1 <- DirichReg(Topics_matrix ~ vegetables.sd + u3talteru + u3csex + u3v_gj,
    data = Nutribiome_Nutr)

model2 <- DirichReg(Topics_matrix ~ vegetables.sd + u3talteru + u3csex + u3v_gj +
    ed2 + u3ttumf, data = Nutribiome_Nutr)

model3 <- DirichReg(Topics_matrix ~ vegetables.sd + u3talteru + u3csex + u3v_gj +
    ed2 + u3ttumf + relevel(u3tcigsmk, ref = "3") + relevel(u3tphys, ref = "2") +
    relevel(u3tmnsaid_reg, ref = "2") + relevel(u3tmmetf, ref = "2"), data = Nutribiome_Nutr)

anova(model_veg2, model_veg3)
# model 2 is significantly better than 1 (p=2.0e-8) model 3 significantly better
# than 2 (p=4.4e-6)


model3q <- update(model3, . ~ . + I(u3talteru^2))
anova(model3, model3q)
# no significant difference when quadratic effect added


# Final Dirichlet model, repeat for each food/nutrient/DQS
model.vegetables <- DirichReg(Topics_matrix ~ vegetables.sd + u3talteru + u3csex +
    u3v_gj + ed2 + relevel(u3tcigsmk, ref = 3) + relevel(u3tphys, ref = 2), data = Nutribiome_Nutr)
```

Then we extract the results of each model for the heat map and combine them into themed matrices.

```
# repeat for each food/nutrient/DQS

model.sum <- (summary(model.vegetables))
coef2 <- model.sum$coef.mat
selected <- grep("vegetables.sd", dimnames(coef2)[[1]])
coefmatrix <- coef2[selected, ]
```

```
estvector <- coefmatrix[, -c(2:4)]
estmatrix <- matrix(estvector, nrow = 1, ncol = 20)
colnames(estmatrix) <- c("Topic_1", "Topic_2", "Topic_3", "Topic_4", "Topic_5", "Topic_6",
    "Topic_7", "Topic_8", "Topic_9", "Topic_10", "Topic_11", "Topic_12", "Topic_13",
    "Topic_14", "Topic_15", "Topic_16", "Topic_17", "Topic_18", "Topic_19", "Topic_20")
rownames(estmatrix) <- c("Vegetables")
estmatrixvegetables <- estmatrix

pvector <- coefmatrix[, -c(1:3)]
pmatrix <- matrix(pvector, nrow = 1, ncol = 20)
colnames(pmatrix) <- c("Topic_1", "Topic_2", "Topic_3", "Topic_4", "Topic_5", "Topic_6",
    "Topic_7", "Topic_8", "Topic_9", "Topic_10", "Topic_11", "Topic_12", "Topic_13",
    "Topic_14", "Topic_15", "Topic_16", "Topic_17", "Topic_18", "Topic_19", "Topic_20")
rownames(pmatrix) <- c("Vegetables")
pmatrixvegetables <- pmatrix


# combining matrices (one for estimate, one for p-value for each group)
nutrmatrixest <- rbind(estmatrixethanol, estmatrixproteing, estmatrixfatg, estmatrixcarbg,
    estmatrixfiberg, estmatrixsolfiber, estmatrixinsfiber)
nutrmatrixp <- rbind(pmatrixethanol, pmatrixproteing, pmatrixfatg, pmatrixcarbg,
    pmatrixfiberg, pmatrixsolfiber, pmatrixinsfiber)
foodmatrixest <- rbind(estmatrixpotatoes, estmatrixvegetables, estmatrixfruit, estmatrixlegumes,
    estmatrixnutsseeds, estmatrixplantoil, estmatrixanimalfat, estmatrixeggs, estmatrixdairy,
    estmatrixcheese, estmatrixyogurt, estmatrixwholegrains, estmatrixrefinedgrains,
    estmatrixfreshredmeat, estmatrixprocessedmeat, estmatrixfish, estmatrixsweets,
    estmatrixcake, estmatrixssb, estmatrixcoffee, estmatrixwine, estmatrixbeer)
foodmatrixp <- rbind(pmatrixpotatoes, pmatrixvegetables, pmatrixfruit, pmatrixlegumes,
    pmatrixnutsseeds, pmatrixplantoil, pmatrixanimalfat, pmatrixeggs, pmatrixdairy,
    pmatrixcheese, pmatrixyogurt, pmatrixwholegrains, pmatrixrefinedgrains, pmatrixfreshredmeat,
    pmatrixprocessedmeat, pmatrixfish, pmatrixsweets, pmatrixcake, pmatrixssb, pmatrixcoffee,
    pmatrixwine, pmatrixbeer)
dpmatrixest <- rbind(estmatrixAHEI, estmatrixMDS)
dpmatrixp <- rbind(pmatrixAHEI, pmatrixMDS)
```

Now we want to find out if there is an association between metabolic diseases or risk factors and the subgroups. Again we use Dirichlet regressions.

First we look at diabetes. We create a variable that includes a 5th category including participants with missings or another type of diabetes and then exclude any participants who were not fasted for the blood draw or with missing covariate information. Then we fit the model and export the results for the heat map.

```
library(forcats)
table(Nutribiome_Topics$dm4, useNA = "always")
Nutribiome_Topics$dm5 <- fct_explicit_na(Nutribiome_Topics$dm4)
table(Nutribiome_Topics$dm5, useNA = "always")

Nutribiome_DM5 <- Nutribiome_Topics[!Nutribiome_Topics$u3tnuecht == 2 & !is.na(Nutribiome_Topics$u3tnue
    !is.na(Nutribiome_Topics$ed2), ]

library("DirichletReg")

Topics_matrix <- DR_data(Nutribiome_DM5[, c("Topic_1", "Topic_2", "Topic_3", "Topic_4",
```

```
    "Topic_5", "Topic_6", "Topic_7", "Topic_8", "Topic_9", "Topic_10", "Topic_11",
    "Topic_12", "Topic_13", "Topic_14", "Topic_15", "Topic_16", "Topic_17", "Topic_18",
    "Topic_19", "Topic_20")], trafo = F)


# Fitting the model
model.topics.DM5 <- DirichReg(Topics_matrix ~ dm5 + u3talteru + u3csex + ed2 + relevel(u3tcigsmk,
    ref = "Never") + relevel(u3tphys, ref = "Inactive"), data = Nutribiome_DM5)



# Exporting results for heat map
model.sum <- (summary(model.topics.DM5))
DMcoef2 <- model.sum$coef.mat

selected <- grep("dm5", dimnames(DMcoef2)[[1]])
coefmatrix <- DMcoef2[selected, ]
estvector <- coefmatrix[, -c(2:4)]
estmatrix <- matrix(estvector, nrow = 4, ncol = 20)
colnames(estmatrix) <- c("Topic_1", "Topic_2", "Topic_3", "Topic_4", "Topic_5", "Topic_6",
    "Topic_7", "Topic_8", "Topic_9", "Topic_10", "Topic_11", "Topic_12", "Topic_13",
    "Topic_14", "Topic_15", "Topic_16", "Topic_17", "Topic_18", "Topic_19", "Topic_20")
rownames(estmatrix) <- c("Prediabetes", "UDM", "PrevalentDM2", "Other")
estmatrixDM <- estmatrix

pvector <- coefmatrix[, -c(1:3)]
pmatrix <- matrix(pvector, nrow = 4, ncol = 20)
colnames(pmatrix) <- c("Topic_1", "Topic_2", "Topic_3", "Topic_4", "Topic_5", "Topic_6",
    "Topic_7", "Topic_8", "Topic_9", "Topic_10", "Topic_11", "Topic_12", "Topic_13",
    "Topic_14", "Topic_15", "Topic_16", "Topic_17", "Topic_18", "Topic_19", "Topic_20")
rownames(pmatrix) <- c("Prediabetes", "UDM", "PrevalentDM2", "Other")
pmatrixDM <- pmatrix
```

Here we do the same for the HDL-c, LDL-c, total cholesterol and total triglyceride models. Since these are all continuous variables, we again standardize them by sd.

```
Nutribiome_Lipids <- Nutribiome_Topics[complete.cases(Nutribiome_Topics$u3lk_ldla) &
    complete.cases(Nutribiome_Topics$u3tmhypol) & complete.cases(Nutribiome_Topics$u3tedyrs) &
    complete.cases(Nutribiome_Topics$u3tnuecht), ]
# 10 removed for missing covariate data, now 1984

Nutribiome_Lipids <- Nutribiome_Lipids[!Nutribiome_Lipids$u3tnuecht == 2, ]
# Total 1972


(sd.HDL <- sd(Nutribiome_Lipids$u3lk_hdln, na.rm = T))
Nutribiome_Lipids$HDL.sd <- Nutribiome_Lipids$u3lk_hdln/sd.HDL

(sd.LDL <- sd(Nutribiome_Lipids$u3lk_ldln, na.rm = T))
Nutribiome_Lipids$LDL.sd <- Nutribiome_Lipids$u3lk_ldln/sd.LDL

(sd.TG <- sd(Nutribiome_Lipids$u3lk_trin, na.rm = T))
Nutribiome_Lipids$TG.sd <- Nutribiome_Lipids$u3lk_trin/sd.TG
```

```r
(sd.TC <- sd(Nutribiome_Lipids$u3lk_choln, na.rm = T))
Nutribiome_Lipids$TC.sd <- Nutribiome_Lipids$u3lk_choln/sd.TC



Topics_matrix <- DR_data(Nutribiome_Lipids[, c("Topic_1", "Topic_2", "Topic_3", "Topic_4",
    "Topic_5", "Topic_6", "Topic_7", "Topic_8", "Topic_9", "Topic_10", "Topic_11",
    "Topic_12", "Topic_13", "Topic_14", "Topic_15", "Topic_16", "Topic_17", "Topic_18",
    "Topic_19", "Topic_20")], trafo = F)


# HDL-c Model
model.topics.HDL <- DirichReg(Topics_matrix ~ HDL.sd + u3talteru + u3csex + ed2 +
    relevel(u3tcigsmk, ref = "Never") + relevel(u3tphys, ref = "Inactive") + relevel(u3tmhypol,
    ref = 2), data = Nutribiome_Lipids)


# LDL-c Model
model.topics.LDL <- DirichReg(Topics_matrix ~ LDL.sd + u3talteru + u3csex + ed2 +
    relevel(u3tcigsmk, ref = "Never") + relevel(u3tphys, ref = "Inactive") + relevel(u3tmhypol,
    ref = 2), data = Nutribiome_Lipids)


# TC Model
model.topics.TC <- DirichReg(Topics_matrix ~ TC.sd + u3talteru + u3csex + ed2 + relevel(u3tcigsmk,
    ref = "Never") + relevel(u3tphys, ref = "Inactive") + relevel(u3tmhypol, ref = 2),
    data = Nutribiome_Lipids)


# TG Model
model.topics.TG <- DirichReg(Topics_matrix ~ TG.sd + u3talteru + u3csex + ed2 + relevel(u3tcigsmk,
    ref = "Never") + relevel(u3tphys, ref = "Inactive") + relevel(u3tmhypol, ref = 2),
    data = Nutribiome_Lipids)


# Exporting results for heat map; repeat for each model

model.sum <- (summary(model.topics.TG))
coef2 <- model.sum$coef.mat
selected <- grep("LDL.sd", dimnames(coef2)[[1]])
coefmatrix <- coef2[selected, ]

estvector <- coefmatrix[, -c(2:4)]
estmatrix <- matrix(estvector, nrow = 1, ncol = 20)
colnames(estmatrix) <- c("Topic_1", "Topic_2", "Topic_3", "Topic_4", "Topic_5", "Topic_6",
    "Topic_7", "Topic_8", "Topic_9", "Topic_10", "Topic_11", "Topic_12", "Topic_13",
    "Topic_14", "Topic_15", "Topic_16", "Topic_17", "Topic_18", "Topic_19", "Topic_20")
rownames(estmatrix) <- c("LDL")
estmatrixLDL <- estmatrix

pvector <- coefmatrix[, -c(1:3)]
pmatrix <- matrix(pvector, nrow = 1, ncol = 20)
colnames(pmatrix) <- c("Topic_1", "Topic_2", "Topic_3", "Topic_4", "Topic_5", "Topic_6",
```

```
    "Topic_7", "Topic_8", "Topic_9", "Topic_10", "Topic_11", "Topic_12", "Topic_13",
    "Topic_14", "Topic_15", "Topic_16", "Topic_17", "Topic_18", "Topic_19", "Topic_20")
rownames(pmatrix) <- c("LDL")
pmatrixLDL <- pmatrix
```

We repeat for BMI and waist circumference, but now only exclude participants with missing covariate info.

```
summary(Nutribiome_Topics$u3tbmi)  #0 NA

Nutribiome_BMI <- Nutribiome_Topics[complete.cases(Nutribiome_Topics$u3tedyrs), ]
# 2 removed for missing covariate data

(sd.BMI <- sd(Nutribiome_BMI$u3tbmi, na.rm = T))
Nutribiome_BMI$BMI.sd <- Nutribiome_BMI$u3tbmi/sd.BMI

(sd.WC <- sd(Nutribiome_BMI$u3ttumf, na.rm = T))
Nutribiome_BMI$WC.sd <- Nutribiome_BMI$u3ttumf/sd.WC




Topics_matrix <- DR_data(Nutribiome_BMI[, c("Topic_1", "Topic_2", "Topic_3", "Topic_4",
    "Topic_5", "Topic_6", "Topic_7", "Topic_8", "Topic_9", "Topic_10", "Topic_11",
    "Topic_12", "Topic_13", "Topic_14", "Topic_15", "Topic_16", "Topic_17", "Topic_18",
    "Topic_19", "Topic_20")], trafo = F)


model.topics.BMI <- DirichReg(Topics_matrix ~ BMI.sd + u3talteru + u3csex + ed2 +
    relevel(u3tcigsmk, ref = "Never") + relevel(u3tphys, ref = "Inactive"), data = Nutribiome_BMI)


model.topics.WC <- DirichReg(Topics_matrix ~ WC.sd + u3talteru + u3csex + ed2 + relevel(u3tcigsmk,
    ref = "Never") + relevel(u3tphys, ref = "Inactive"), data = Nutribiome_BMI)


# exporting results for heat map #

# repeat for BMI and waist circumference models
model.sum <- (summary(model.topics.BMI))
coef2 <- model.sum$coef.mat
selected <- grep("BMI.sd", dimnames(coef2)[[1]])
coefmatrix <- coef2[selected, ]

estvector <- coefmatrix[, -c(2:4)]
estmatrix <- matrix(estvector, nrow = 1, ncol = 20)
colnames(estmatrix) <- c("Topic_1", "Topic_2", "Topic_3", "Topic_4", "Topic_5", "Topic_6",
    "Topic_7", "Topic_8", "Topic_9", "Topic_10", "Topic_11", "Topic_12", "Topic_13",
    "Topic_14", "Topic_15", "Topic_16", "Topic_17", "Topic_18", "Topic_19", "Topic_20")
rownames(estmatrix) <- c("BMI")
estmatrixBMI <- estmatrix

pvector <- coefmatrix[, -c(1:3)]
pmatrix <- matrix(pvector, nrow = 1, ncol = 20)
colnames(pmatrix) <- c("Topic_1", "Topic_2", "Topic_3", "Topic_4", "Topic_5", "Topic_6",
```

```
    "Topic_7", "Topic_8", "Topic_9", "Topic_10", "Topic_11", "Topic_12", "Topic_13",
    "Topic_14", "Topic_15", "Topic_16", "Topic_17", "Topic_18", "Topic_19", "Topic_20")
rownames(pmatrix) <- c("BMI")
pmatrixBMI <- pmatrix
```

And again for hypertension.

```
table(Nutribiome_Topics$u3thycont, useNA = "always")  #1 NA
table(Nutribiome_Topics$u3tantihy, useNA = "always")  #anti-hypertensives #0 NA

Nutribiome_HTN <- Nutribiome_Topics[complete.cases(Nutribiome_Topics$u3thycont) &
    complete.cases(Nutribiome_Topics$u3tedyrs), ]
# 1989 observations


Topics_matrix <- DR_data(Nutribiome_HTN[, c("Topic_1", "Topic_2", "Topic_3", "Topic_4",
    "Topic_5", "Topic_6", "Topic_7", "Topic_8", "Topic_9", "Topic_10", "Topic_11",
    "Topic_12", "Topic_13", "Topic_14", "Topic_15", "Topic_16", "Topic_17", "Topic_18",
    "Topic_19", "Topic_20")], trafo = F)

model.topics.HTN5o <- DirichReg(Topics_matrix ~ relevel(u3thycont, ref = 5) + u3talteru +
    u3csex + ed2 + relevel(u3tcigsmk, ref = "Never") + relevel(u3tphys, ref = "Inactive"),
    data = Nutribiome_HTN)


# extracting values for heat map #
model.sum <- (summary(model.topics.HTN5o))
coef2 <- model.sum$coef.mat
selected <- grep("u3thycont", dimnames(coef2)[[1]])
coefmatrix <- coef2[selected, ]
estvector <- coefmatrix[, -c(2:4)]
estmatrix <- matrix(estvector, nrow = 4, ncol = 20)
colnames(estmatrix) <- c("Topic_1", "Topic_2", "Topic_3", "Topic_4", "Topic_5", "Topic_6",
    "Topic_7", "Topic_8", "Topic_9", "Topic_10", "Topic_11", "Topic_12", "Topic_13",
    "Topic_14", "Topic_15", "Topic_16", "Topic_17", "Topic_18", "Topic_19", "Topic_20")
rownames(estmatrix) <- c("Controlled_HTN", "Uncontrolled_HTN", "Untreated_HTN", "Unknown_HTN")
estmatrixHTN <- estmatrix

pvector <- coefmatrix[, -c(1:3)]
pmatrix <- matrix(pvector, nrow = 4, ncol = 20)
colnames(pmatrix) <- c("Topic_1", "Topic_2", "Topic_3", "Topic_4", "Topic_5", "Topic_6",
    "Topic_7", "Topic_8", "Topic_9", "Topic_10", "Topic_11", "Topic_12", "Topic_13",
    "Topic_14", "Topic_15", "Topic_16", "Topic_17", "Topic_18", "Topic_19", "Topic_20")
rownames(pmatrix) <- c("Controlled_HTN", "Uncontrolled_HTN", "Untreated_HTN", "Unknown_HTN")
pmatrixHTN <- pmatrix
```

Here we combine all of the disease/risk factor results into two matrices, one for the estimates and one for the p values.

```
diseasematrixest <- rbind(estmatrixBMI, estmatrixWC, estmatrixHDL, estmatrixLDL,
    estmatrixTC, estmatrixTG, estmatrixDM, estmatrixHTN)
diseasematrixp <- rbind(pmatrixBMI, pmatrixWC, pmatrixHDL, pmatrixLDL, pmatrixTC,
    pmatrixTG, pmatrixDM, pmatrixHTN)
```

Now that we have all results, we can create the heat map. This is done according to Haarman et al.(2015) (https://doi.org/10.1016/j.jbi.2014.10.003). This section of our code has been adapted from the code published with their respective manuscript.

We create one matrix containing the estimates for all models and another matrix containing the p values. Finally, three plots are produced. One showing the effect size for each item (color), one displaying the significance (circle size), and one indicating which associations are significant after adjustment (small dot). These plots were then combined using Inkscape.

```
load("legend_significance.rda")  #from Haarman et al. manuscript (link above)

plot_size <- rbind(foodmatrixest, nutrmatrixest, dpmatrixest, diseasematrixest)
# matrix of estimates

plot_significance <- rbind(foodmatrixp, nutrmatrixp, dpmatrixp, diseasematrixp)
# matrix of p values

# transforming for circle size for proper proportions - Haarman et al.
plot_significancet <- sapply(plot_significance, function(x) 1 - (x^(1/3)))
plot_significancet <- matrix(plot_significancet, nrow = 45, ncol = 20)
rownames(plot_significancet) <- rownames(plot_significance)
colnames(plot_significancet) <- colnames(plot_significance)


library(corrplot)

# Define colors
col <- colorRampPalette(c("royalblue4", "royalblue2", "royalblue1", "White", "orangered1",
    "orangered2", "orangered4"), interpolate = "linear")

# Create significance plot
corrplot(plot_significancet, method = c("circle"), col = ("black"), tl.cex = 0.6,
    tl.col = ("black"), cl.pos = "n", addgrid.col = NA, outline = F)

# Create effect size plot
corrplot(plot_size, is.corr = FALSE, method = c("color"), addgrid.col = NA, col = col(200),
    tl.cex = 0.6, tl.col = "black", cl.pos = "n")

# Create Bonferonni dot plot
corrplot(plot_significance * 0 + 0.05, method = c("circle"), addgrid.col = NA, col = ("black"),
    tl.cex = 0.6, tl.col = ("black"), p.mat = plot_significance, insig = "blank",
    sig.level = 0.00133, cl.pos = "n")

# Create plots with legends
corrplot(legend_significance, method = c("circle"), col = ("grey"), tl.cex = 0.6,
    tl.col = ("black"), cl.pos = "n", add = T)
# used 'legend significance file from Haarman et al. 2015

corrplot(plot_size, is.corr = FALSE, method = c("color"), addgrid.col = NA, col = col(200),
    tl.cex = 0.6, tl.col = "black", cl.pos = "r", cl.lim = c(-0.45, 0.45), cl.ratio = 0.4,
    cl.length = 7, add = T)
```

We then conduct a sub-analysis of the percentage of arrhythmic OTUs (as identified by Reitmeier et al. (2019) (https://doi.org/10.1101/2019.12.27.889865).

```r
ArrhythmicOTUs <- read.xlsx(file = "ArrhythmicOTUs.xlsx", 1, header = F)
# all arrhythmic OTUs (87)

DiabetesOTUs <- read.xlsx(file = "ArrhythmicOTUs.xlsx", 2, header = F)
# diabetes-specific OTUs (14)

ObesityOTUs <- read.xlsx(file = "ArrhythmicOTUs.xlsx", 3, header = F)
# obesity-specific OTUs (51)


# percentages for all arrhythmic OTUs in all topics
rownames(ArrhythmicOTUs) <- ArrhythmicOTUs$X1
ArrhythmicOTUsv <- rownames(ArrhythmicOTUs)
subsettedmybeta <- mybeta_df[ArrhythmicOTUsv]
ArrhythmicOTUsums <- as.data.frame(rowSums(subsettedmybeta))

# percentages for all diabetes risk OTUs in all topics
rownames(DiabetesOTUs) <- DiabetesOTUs$X1
DiabetesOTUsv <- rownames(DiabetesOTUs)
subsettedmybetadm <- mybeta_df[DiabetesOTUsv]
DiabetesOTUsums <- as.data.frame(rowSums(subsettedmybetadm))

# percentages for all obesity-specific OTUs in all topics
rownames(ObesityOTUs) <- ObesityOTUs$X1
ObesityOTUsv <- rownames(ObesityOTUs)
subsettedmybetaobesity <- mybeta_df[ObesityOTUsv]
ObesityOTUsums <- as.data.frame(rowSums(subsettedmybetaobesity))
```
